# Combined *SEPT9* and *BMP3* methylation in plasma for colorectal cancer early detection and screening in a Brazilian population

**DOI:** 10.1002/cam4.6224

**Published:** 2023-06-20

**Authors:** Adhara Brandão Lima, Mariana Bisarro dos Reis, Marcus Matsushita, Monise Tadin dos Reis, Marco Antônio de Oliveira, Rui Manuel Reis, Denise Peixoto Guimarães

**Affiliations:** ^1^ Molecular Oncology Research Center Barretos Cancer Hospital Barretos Brazil; ^2^ Department of Pathology Barretos Cancer Hospital Barretos Brazil; ^3^ Nucleous of Epidemiology and Statistics Barretos Cancer Hospital Barretos Brazil; ^4^ Life and Health Sciences Research Institute (ICVS), Medical School University of Minho Braga Portugal; ^5^ ICVS/3B's‐PT Government Associate Laboratory Braga Portugal; ^6^ Department of Endoscopy Barretos Cancer Hospital Barretos Brazil

**Keywords:** cell‐free DNA, colorectal cancer, digital PCR, DNA methylation, liquid biopsy

## Abstract

**Background:**

Colorectal cancer (CRC) screening can help to reduce its incidence and mortality. Noninvasive strategies, such as plasma analysis of epigenetic alterations, can constitute important biomarkers of CRC detection.

**Objective:**

This study aimed to evaluate the plasma methylation status of *SEPT9* and *BMP3* promoters as biomarkers for detection of CRC and its precursor lesions in a Brazilian population.

**Methods:**

Plasma samples from 262 participants of the CRC screening program of Barretos Cancer Hospital who had a positive fecal occult blood test and underwent colonoscopy and cancer patients were analyzed. Participants were grouped according to the worst lesion detected in the colonoscopy. Cell‐free circulating DNA (cfDNA) was bisulfite treated followed by the analysis of *SEPT9* and *BMP3* methylation status using a droplet digital PCR system (ddPCR). The best methylation cutoff value for group discrimination was calculated by receiver operating characteristic (ROC) curve analysis.

**Results:**

Among the 262 participants, 38 were diagnosed with CRC, 46 with advanced adenomas 119 with nonadvanced adenomas, three with sessile serrated lesions, and 13 with hyperplastic polyps. In 43 participants, no lesion was detected in the colonoscopy and were used as controls. The CRC group showed the highest cfDNA concentration (10.4 ng/mL). For the *SEPT9* gene, a cutoff of 2.5% (AUC = 0.681) that discriminates between CRC and the control group resulted in CRC sensitivity and specificity of 50% and 90%, respectively. Concerning the *BMP3* gene, a cutoff of 2.3% (AUC = 0.576) showed 40% and 90% of sensitivity and specificity for CRC detection, respectively. Combining *SEPT9*, *BMP3* status, and age over 60 years resulted in a better performance for detecting CRC (AUC = 0.845) than the individual gene models, yielding 80% and 81% of sensitivity and specificity, respectively.

**Conclusion:**

The present study suggests that a combination of *SEPT9* and *BMP3* plasma methylation, along with age over 60 years, showed the highest performance in detecting CRC in a Brazilian population. These noninvasive biomarkers can potentially serve as useful tools for CRC screening programs.

## 1 INTRODUCTION

Colorectal cancer (CRC) is the second leading cause of cancer‐related death worldwide.^1^ According to GLOBOCAN, over 1,9 million new CRC cases, and 935.173 deaths were estimated to occur in 2020.^1^ In Brazil, CRC ranks second in incidence among women and men^2^ with over 45.630 cases estimated annually in the 2023–25 triennium.^2^ In addition, CRC is often diagnosed at advanced stages, resulting insignificantly worse outcomes, with only 12% 5‐year relative survival for metastatic disease (Stage IV CRC).^3^ Thus, accurate screening methods for precursor lesions or early‐stage CRC detection are urgently needed.^4^

The majority (89%–90%) of CRC cases develop through the adenoma‐carcinoma sequence, while 10%–20% develop via the serrated pathway, with adenoma and sessile serrated lesion (SSL) being the main precursor lesions, respectively.^5^, ^6^ As CRC typically progresses over a long period of 7–10 years from adenoma to carcinoma, it is a highly suitable screening disease.^7^ International guidelines have thus recommended CRC screening (using colonoscopy or fecal tests) to reduce CRC incidence and mortality for populations where CRC is frequent.^8^ Although colonoscopy is highly sensitive, its invasiveness and high costs are significant disadvantages. Inversely, fecal tests are a noninvasive alternative, but have a lower adenoma detection capacity.^9^ A liquid biopsy‐based strategy for isolating circulating biomarkers (cell‐free DNA, tumor cells, tumor DNA) from body fluids provides a noninvasive opportunity for CRC early diagnosis.^10^ Aberrant DNA methylation occurs frequently during CRC development.^11^, ^12^, ^13^ Higher methylated *SEPT9* (mSEPT9) levels have been found in blood from CRC cases and in CRC tissue samples compared to normal colonoscopy biopsy samples or blood from healthy individuals.^15^, ^16^, ^17^ These results led to the Epi proColon test (Epigenomics, USA), a blood‐based FDA‐approved screening test for adults with average risk for CRC, which evaluates *SEPT9* methylation levels in a quantitative assay based on real‐time PCR reaction.^14^ The Cologuard® (Exact Sciences), another FDA‐approved noninvasive commercialized test in liquid biopsy (feces), is a quantitative assay that includes methylation of *BMP3* and *NDRG4* genes, mutations of oncogene *KRAS*, and immunochemical fecal occult blood test (FIT).^9^ Although Cologuard® has a higher sensitivity to detect advanced adenomas (AA) than the immunochemical fecal test alone, it had significant false‐positive results.^18^, ^19^

Nevertheless, there are several challenges that need to be addressed, including the low capacity for adenoma detection, significant discrepancies in the diagnostic accuracy for CRC across studies of *SEPT9* test,^20^, ^21^, ^22^, ^23^, ^24^ and lower CRC specificity of Cologuard® for CRC compared to FIT. Moreover, it is crucial to validate these biomarkers in distinct populations, particularly in underrepresented genomic and epigenomic studies, such as the South‐American.^25^

Our group recently demonstrated higher DNA methylation levels for both *SEPT9* and *BMP3* genes in CRC tumor tissue compared to normal tissue in a Brazilian population.^15^ In the present study, we investigated the role of cell‐free circulating DNA (cfDNA) methylation analysis of *SEPT9* and *BMP3* genes in the plasma of CRC screening program participants for detecting both adenomas and CRC lesions.

## 2 METHODS

### 2.1 Population

**FIGURE 1:**
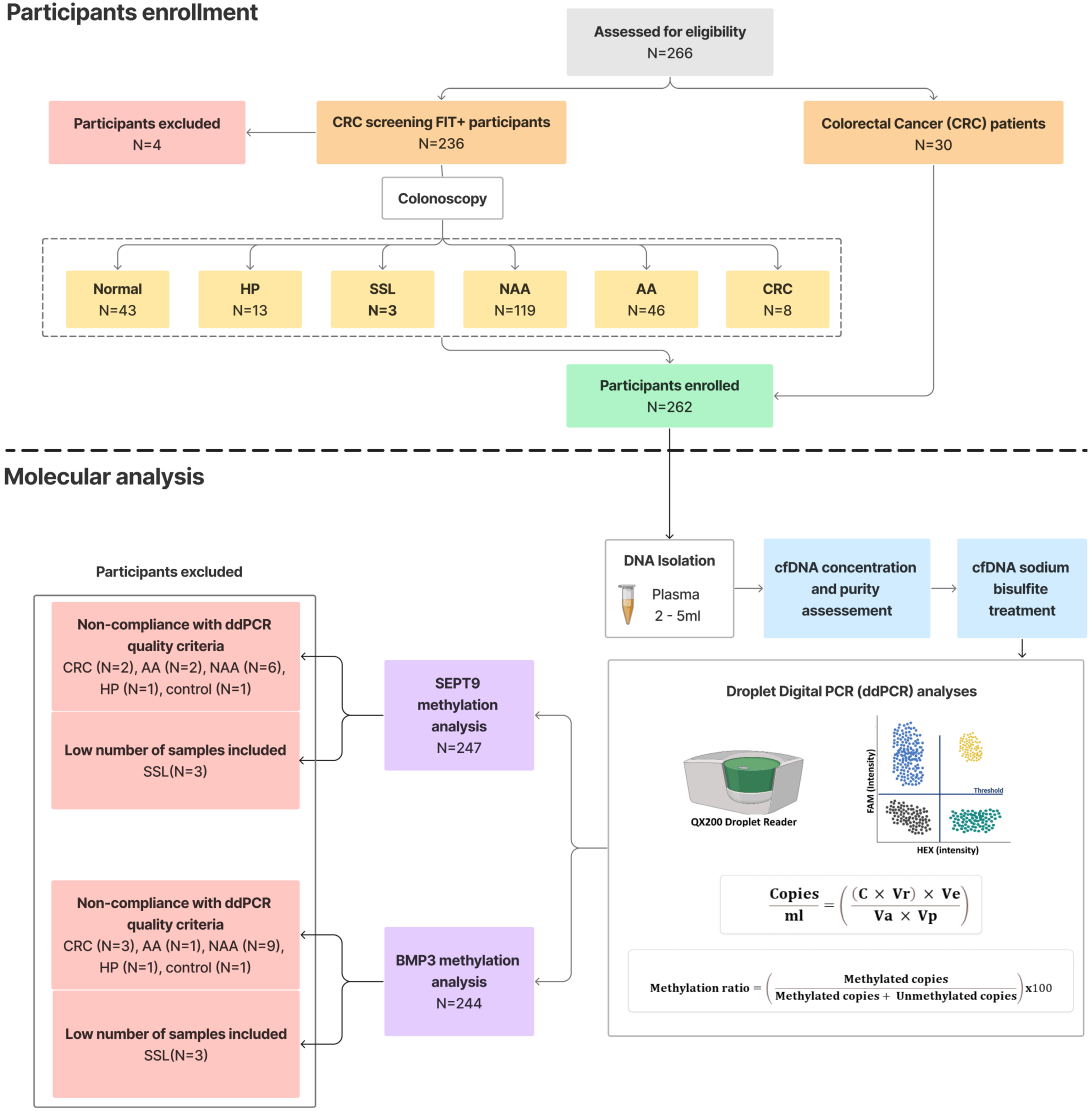
Flowchart of the study design. AA, advanced adenoma; cfDNA, cell‐free DNA; CRC, colorectal cancer; HP, hyperplastic polyp; NAA, nonadvanced adenoma; SSL, sessile serrated lesion.

From 2016 to 2019, a total of 262 participants were enrolled, being 232 participants of Barretos Cancer Hospital screening CRC program, who had follow‐up colonoscopy after positive FIT test.^26^ The remaining 30 participants enrolled were CRC diagnosed at the Barretos Cancer Hospital (BCH). Exclusion criteria were as follows: (1) the presence of other significant medical history including any type of cancer, inflammatory bowel diseases, and hereditary cancer syndromes; (2) blood collection performed after colonoscopy or surgery. A flowchart of the study design is showed in Figure 1.

Detected lesions were endoscopically classified according to the Paris classification.^27^ The histological evaluation of each lesion was performed based on the World Health Organization (WHO) tumors 2019 classification.^5^ The AA classification was based on the following criteria: (1) at least 10 mm in diameter; or (2) at least 25% of villous architecture; or (3) high‐grade dysplasia. The staging of CRC was determined using the American Joint Committee on Cancer (AJCC) 8th edition TNM staging system.^28^ Patients were stratified according to the most advanced lesion: adenocarcinoma, AA, nonadvanced adenoma (NAA), SSL, and hyperplastic polyp (HP).

### 2.2 Circulating cell‐free DNA (cfDNA) isolation and bisulfite treatment

Peripheral blood samples were collected in EDTA tubes (BD vacutainer®) and after centrifugation, the plasma aliquots were stored at −80°C at the Barretos Cancer Hospital Biobank^29^ until cfDNA isolation. The plasma was separated from the cellular fraction by centrifugation at 16.000× *g* for 10 min at 4°C and cfDNA was isolated using 2–5 mL as starting volume of plasma (5 mL was used whenever available).

The cfDNA was isolated using the QIAmp circulating Nucleic Acid Kit (Qiagen) according to the manufacturer's instructions and then eluted in 30 μL. cfDNA concentration was determined by Qubit™ dsDNA High Sensitivity Assay (Thermo Fisher Scientific) with a total volume of 1 μL of cfDNA and normalized for plasma volume: cfDNA concentration in ng/mL of plasma = (cfDNA quantification in ng/μL × elution volume in μL)/plasma volume in mL. cfDNA purity and fragment size was assessed using TapeStation 2200 (Agilent®) with a High Sensitivity D1000 Screen Tapesystem (Agilent®) by using a total volume of 2 μL of cfDNA. The cfDNA was stored at −80°C until analysis.

Isolated cfDNA was sodium bisulfite treated using EZ DNA Methylation‐Gold Kit (Zymo Research) following the manufacturer's protocols. The cfDNA obtained, contained small fragments of around 150–200 bp as well as larger fragments in agreement with a previous report^30^ (Figure S1).

### 2.3 Primers and probes design

Primers and probes for *SEPT9* and *BMP3* gene were designed to amplify a region containing CpG sites located in in the first exon/promoter regions of *SEPT9* transcript v2 (same region evaluated by the Epi proColon® assay) and promoter region of *BMP3*. The overlapping CpG islands of both genes were based on sequences obtained from USCS Genome Browser (GRCh38/hg38human assembly‐https://genome.ucsc.edu/cgi‐bin/hgGateway). FAM and HEX‐labeled probes were specific for methylated and unmethylated CpG sites in the promoter, respectively (Table S1).^31^, ^32^ These regions were previously analyzed by our group and found to be highly methylated in CRC tissue.^15^ Additionally, we used the OligoAnalyzer Tool (https://www.idtdna.com/calc/analyzer) to assess various properties of each sequence, such as GC content, melting temperature, hairpins, dimers, and mismatches.

### 2.4 Droplet Digital PCR (ddPCR)

A standard mutation/SNP singleplex assay strategy was used to quantify methylation targets in ddPCR analyses based on bisulfite converted DNA samples. The methylation status was calculated as the ratio between methylated and unmethylated DNA.^33^

Droplet generation was performed by an automated droplet generator (Bio‐Rad). The ddPCR reaction mixture was done in a final volume of 20 μL, consisting of 2x ddPCR Supermix for Probes (no dUTP) (Bio‐Rad), 900 nM of each primer, 250 nM of the probe, and up to 10 ng bisulfite‐converted DNA template (the input volume varied from 5 to 5.94 μL of bisulfite‐converted DNA). Droplets were generated in the Automated Droplet Generator (Bio‐Rad) and amplification performed using a C1000 Touch™ Thermal Cycle 96‐Deep Well Reaction Module (Bio‐Rad) (Figure S2) and conditions were as follows: pre heating for DNA polymerase activation at 95°C for 10 min followed by 39 cycles of 94°C for 30 s denaturation, 47°C (*SEPT9*) or 50°C (*BMP3*) for 60 s annealing and extension, a final heating at 98°C for 10 min. The temperature ramp rate was set to 2.5°C/s, with the lid heat of 105°C, according to the Bio‐Rad recommendations. We conducted temperature gradient experiments for *SEPT9* (ranging from 46 to 51°C) and *BMP3* (ranging from 48.8 to 59.1°C) to determine the optimal annealing temperature for maximizing the separation between droplets (Figure S3 and S4).

Methylation‐positive control (Zymo Research), methylation‐negative control (Zymo Research) (a DNA mixture consisting of 50% methylated and 50% unmethylated DNA was used, with a total amount of 10 ng of DNA in each well) and a non‐template control (NTC) were used in each experiment.^34^ Samples were read in a QX200™ Droplet Reader (Bio‐Rad) (Figure S2). The number of droplets per reaction was determined using a QX‐200 droplet reader and analysis was performed on QuantaSoft software (Bio‐Rad).

### 2.5 Data analysis and determination of limit of blank (LoB) and limit of detection (LoD)

Fluorescence amplitude signals were measured by the software package QuantaSoft v1.7.4 (BioRad). The exclusion criteria for further analysis was a low number of droplets measured (< 10,000 per 20 μL PCR). The data from the ddPCR are given in target copies/μL reaction.

To determine analytical parameters, we calculated the Limit of Blank (LoB), Limit of Detection (LoD), and Limit of Quantification (LoQ) based on Clinical and Laboratory Standards Institute (CLSI) guidelines.^35^ The LoB is the lowest level of methylated/unmethylated DNA that can be reliably detected above the background noise in blank samples (containing no target analyte) (Table S2). The LoB experiment was performed for both methylated and unmethylated probes, to determine false positive rates for each assay. A total of 20 replicates of ddPCR reaction containing unmethylated or methylated probes was used to calculated LoB. LoB was calculated as: mean blank + 1.645*SD blank.^35^, ^36^ For *SEPT9* assay, the LoB was 0.93 copies/reaction for methylated probe and 1.15 copies/reaction for unmethylated probe (Figure S5). For *BMP3* assay, the LoB was 0.94 copies/reaction for methylated probe and 30.04 copies/reaction for unmethylated probe (Figure S6).

After establishing the LoB values, LoD was determined by serial dilutions of the 100% methylated and unmethylated DNA (Human Methylated and Non‐Methylated DNA–Zymo Research). Hypermethylated and unmethylated DNA controls were 10‐fold serially diluted (10 to 0.01 ng) in ultrapure water to test the lower limit of hypermethylated and unmethylated DNA detection. We calculated the lowest analyte concentration that can be measured with 95% confidence from the LoB value, where: LoD = LoB + 1.645*SD low concentration sample.^35^, ^36^ The lowest concentration sample dilution was used to determine the LoD of assays. For *SEPT9* assay, the LoD was 2.22 copies/reaction for methylated probe and 3.48 copies/reaction for unmethylated probe (Figure S7). For *BMP3* assay, the LoD was 2.68 copies/reaction for methylated probe and the unmethylated probe had a LoD of 32.68 copies/reaction (Figure S8). The LoQ was set as the lowest concentration of methylated DNA that can be accurately measured with a coefficient of variation (CV) ≤30%.^35^, ^36^ For *SEPT9* assay, the LoQ was 4.79 copies/reaction for methylated probe and 3.62 copies/reaction for unmethylated probe. For *BMP3* assay, the LoQ was 25.66 copies/reaction for methylated probe and the unmethylated probe had a LoQ of 46.72 copies/reaction (Figure S9).

An external calculation was performed to convert target copies/reaction into copies/mL plasma, where C = copies/reaction; Vr = ddPCR reaction volume, Ve = elution volume; Va = cfDNA volume added into reaction; Vp = Plasma volume:

$$\;\;\mathrm{Copies}\;per\;mL\;plasma\;=\;\frac{1}{V_{a}\;\times \;V_{p\;}}\;\times \;\left(C\times V_{r}\right)\;\times \;V_{e}\;\;\;\;\;\;$$

Methylation status was determined by calculating the ratio of normalized methylated copies to the total number (methylated and unmethylated) of copies,^33^ as following:

$$\begin{array}{c} \text{Methylation ratio}\\ =\left(\frac{\text{Methylated copies}}{\text{Methylated copies}+\text{Unmethylated copies}}\right)\times 100 \end{array}$$

### 2.6 Statistical analysis

R version 4.0.5 and IBM SPSS Statistics for Windows, Version 23.0 (IBM) were used. The *p* < 0.05 was considered statistically significant. Continuous variables difference between groups were compared by using two‐sided Kruskal–Wallis or Mann–Whitney *U*‐test, where appropriated. Categorical variables were described with number (percentages). The imprecision calculation was performed using the R “VFP” package (v1.4.1). We used “cutpointr” R package to calculate the optimal cutoff points of ROC curves. We applied Euclidean's index metric in which the cutoff value corresponds to the point on the receiver operating characteristic curve (ROC) that is closest to the left‐hand corner of ROC space, defined by Distance^2^ = (1 − sensitivity)^2^ + (1 − specificity)^2^. Euclidian's index determined the cutoffs methylation level of both *SEPT9* and *BMP3* gene promoters. Assay performance parameters (sensitivity, specificity, ROC curves, and area under the curve–AUC) were calculated using CRC samples, AAs and controls. The figures were generated using GraphPad Prism for Windows 8.0.1.

## 3 RESULTS

### 3.1 Characteristics of study participants

Table 1 displays the participant age and sex distributions as well as the characteristics of lesions for each group. Among the 262 participants enrolled, 165 (63.0%) were women, with 58 ± 7.0 years old. A total of 38 CRC cases, 46 AAs, 119 nonadvanced adenomas (NAA), three SSLs, and 13 hyperplastic polyps (HP) were included (Table 1). In 43 participants, no lesion was detected on colonoscopy and was used as a control group. Regarding the serrated polyps, 69.2% of the hyperplastic polyps (HP) were found to be located distal to the splenic flexure, whereas 66.7% of the SSLs were located at or proximal to the splenic flexure. Of the AAs, 76.0% were 10 mm or greater in size, and 34.8% exhibited high‐grade dysplasia. Among the CRCs, 73.7% were located distally, being nine (23.7%) Stage I, nine (23.7%) Stage II, 14 (36.8%) Stage III, and six (15%) Stage IV (Table 1).

**TABLE 1:** Demographic data and features of lesions for each group of participants.

	Control^a^ (*n* = 43)	Serrated polyps (*n* = 16)		Adenoma (*n* = 165)		Cancer (*n* = 38)
		SSL (*n* = 3)	HP (*n* = 13)	NAA (*n* = 119)	AA (*n* = 46)	
Age						
Mean (SD)	57.1 (4.4)	56.6 (5.0)	57.6 (4.8)	57.5 (4.4)	58.1 (5.0)	63.8 (13.0)
Sex						
Female	32 (74.4%)	2 (66.7%)	6 (46.2%)	79 (66.4%)	30 (65.2%)	16 (42.1%)
Male	11 (25.6%)	1 (33.3%)	7 (53.8%)	40 (33.6%)	16 (34.8%)	22 (57.9%)
Localization						
Proximal	‐	2 (66.7%)	1 (7.7%)	58 (48.8%)	15 (32.6%)	10 (26.3%)
Distal	‐	0	9 (69.2%)	22 (18.5%)	25 (54.3%)	28 (73.7%)
Both	‐	1 (33.3%)	3 (23.1%)	39 (32.7%)	6 (13.1%)	0
Size						
<10 mm	‐	2 (66.7%)	13 (100%)	119 (100%)	11 (24.0%)	‐
≥10 mm	‐	1 (33.3%)	‐	‐	35 (76.0%)	‐
Dysplasia						
Low‐grade	‐	‐	‐	119 (100%)	30 (65.2%)	‐
High‐grade	‐	‐	‐	‐	16 (34.8%)	‐
Stage						
I	‐	‐	‐	‐	‐	9 (23.7%)
II	‐	‐	‐	‐	‐	9 (23.7%)
III	‐	‐	‐	‐	‐	14 (36.8%)
IV	‐	‐	‐	‐	‐	6 (15.8%)

Abbreviations: AA, advanced adenoma; HP, hyperplastic polyp; NAA, nonadvanced adenoma; SD, standard deviation; SSL, sessile serrated lesion.

^**a**^ Samples with no lesion identified on the colonoscopy.

### 3.2 Circulating (cfDNA) levels in plasma

The present study investigated the circulating levels of cell‐free DNA (cfDNA) in plasma samples obtained from 262 participants. The cfDNA integrity (evaluated by Tapestation), showed a predominant fraction of cfDNA ranging from 150 to 200 bp, in line with previous reports.^30^ Notably, fragments longer than 700 bp were no detectable, indicating the absence of genomic DNA contamination.

**FIGURE 2:**
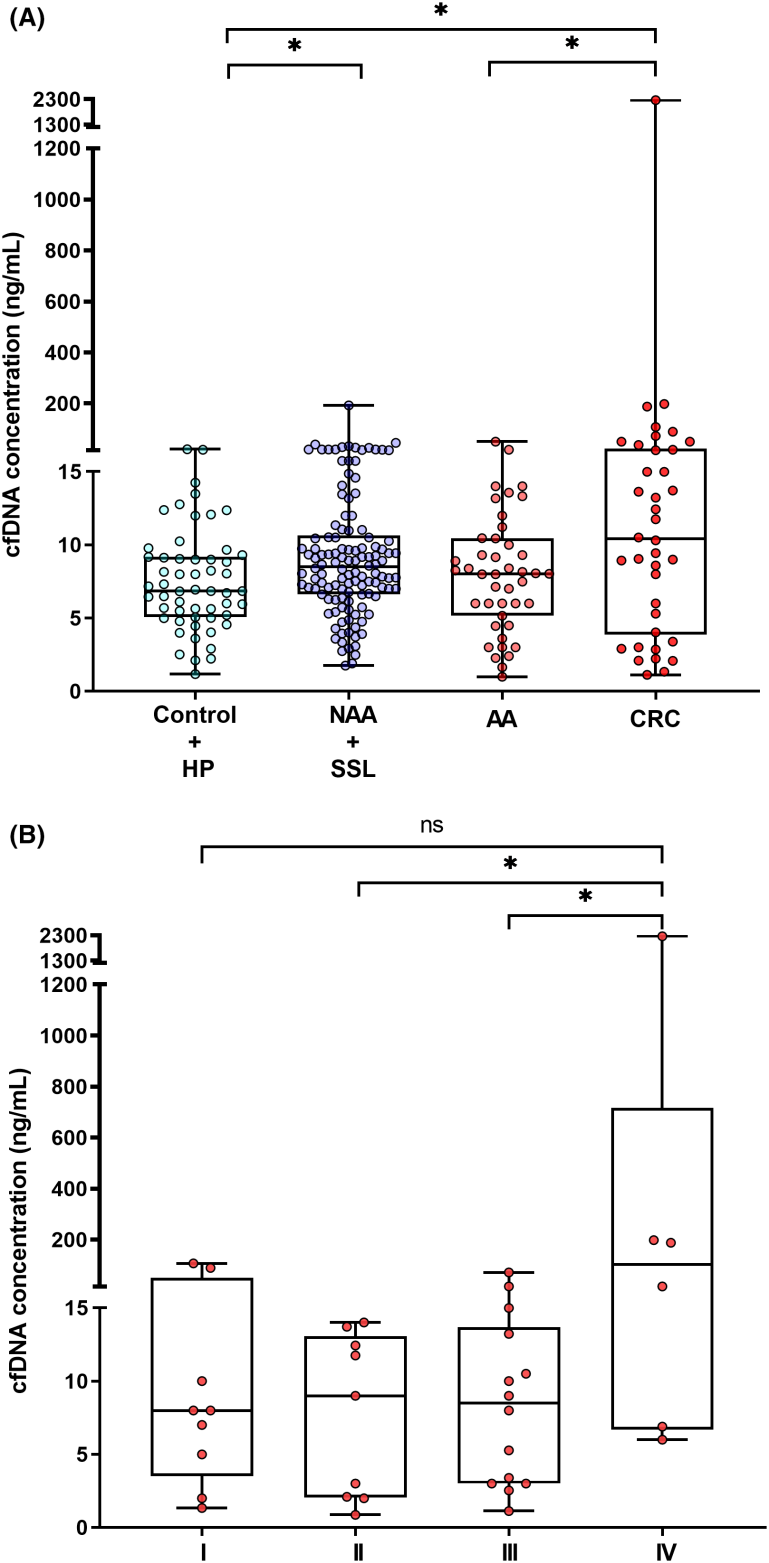
cfDNA concentration (ng/mL) according to: (A) Lesion group (Kruskal–Wallis test; *p* = 0.030) and (B) Tumor stage (I–IV). (Kruskal–Wallis test; *p* = 0.049). Mann–Whitney *U*‐test to compare two groups; * *p* < 0.05. AA, advanced adenoma; cfDNA, cell‐free DNA;CRC, colorectal cancer; HP, hyperplastic polyp; NAA, nonadvanced adenoma; ns, not significant; SSL, sessile serrated lesion.

After normalizing the cfDNA levels by plasma volume, the median concentration was 8.5 ng/mL of plasma (range: 1–2268 ng/mL). The median cfDNA levels (in ng/mL) in each group are presented in Table 2 and Figure 2. The highest cfDNA levels were observed in the NAA + SSL group (8.4 ng/mL) and CRC group (10.4 ng/mL) compared to control + HP group (6.8 ng/mL) (*p* = 0.012 and *p* = 0.019, respectively). The median circulating cfDNA level was also significantly higher in CRC compared to the AA group (8.2 ng/mL; *p* = 0.047) (Figure 2A).

**TABLE 2:** Methylated copies/mL of plasma and methylation status of *SEPT9* and *BMP3*.

Groups	*N*	cfDNA ng/mL median (min–max)	Copies/mL of plasma median (min–max)		Methylation status median (min–max)
			Methylated	Unmethylated	
*SEPT9*					
Control/HP	54	7 (1–29)	4 (0–43)	427 (34–1777)	0% (0–3)
NAA	113	8 (4–192)	3 (0–170)	412 (2–12,556)	1% (0–12)
AA	44	8 (4–50)	4 (0–64)	562 (37–2353)	1% (0–4)
CRC	36	10 (1–2268)	16 (0–23,232)	567 (15–107,490)	2% (0–83)
I	9	9 (1–106)	5 (0–79)	444 (208–5236)	1% (0–5)
II	8	10 (1–14)	5 (0–271)	391 (121–1191)	2% (0–54)
III	13	9 (1–71)	16 (0–255)	451 (69–1434)	2% (0–30)
IV	6	100 (4–2268)	393 (136–23,232)	523 (108–107,049)	67% (20–83)
*BMP3*					
Control/HP	54	7 (1–29)	0 (0–20)	210 (48–1666)	0% (0–11)
NAA	110	8 (4–192)	0 (0–42)	300 (47–4992)	0% (0–13)
AA	45	8 (4–50)	0 (0–10)	234 (36–1131)	0% (0–10)
CRC	35	10 (1–2268)	0 (0–725)	239 (51–183,708)	0% (0–84)
I	9	9 (1–106)	3 (0–57)	444 (208–5236)	4% (0–61)
II	8	10 (1–14)	0 (0–14)	391 (121–1191)	0% (0–63)
III	12	9 (1–71)	0 (0–81)	417 (69–1434)	0% (0–43)
IV	6	100 (4–2268)	20 (0–725)	523 (108–107,049)	6% (0–84)

Abbreviations: AA, advanced adenoma; CRC, colorectal Cancer; HP, hyperplastic polyp; N, number of cases analyzed; NAA, nonadvanced adenoma; SSL, sessile serrated lesion.

Furthermore, we analyzed the median cfDNA levels for each tumor staging group (Table 2 and Figure 2B). The median circulating cfDNA level was significantly higher in the CRC stage IV compared to stage II (*p* = 0.049) and stage III (*p* = 0.043) (Figure 2B).

### 3.3 Association between *SEPT9* and *BMP3* methylation status with participant groups

**FIGURE 3:**
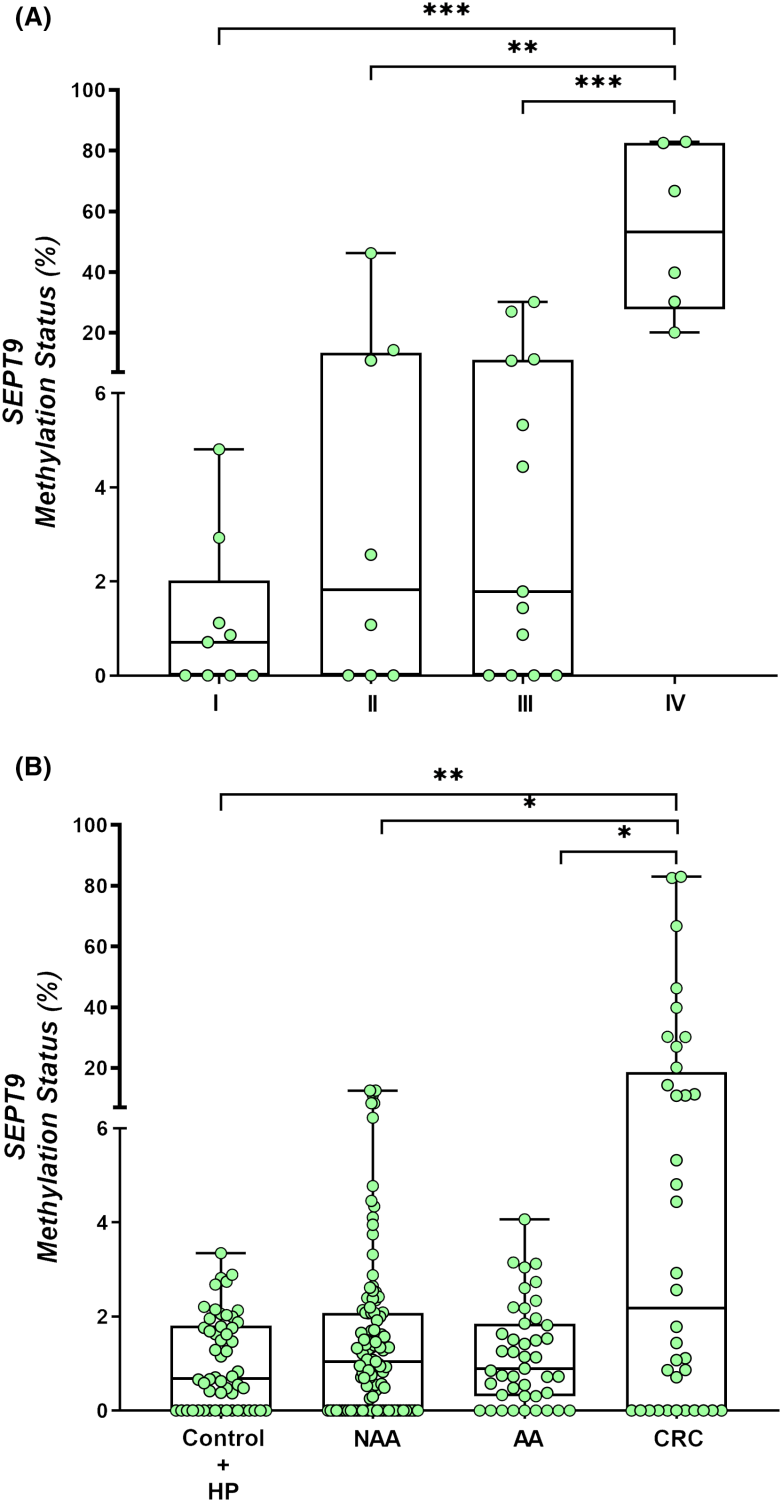
Methylation status of *SEPT9* according to: (A) Lesion group (Kruskal–Wallis test; *p* = 0.047) and (B) Tumor stage (I–IV) (Kruskal–Wallis test; *p* = 0.001). Mann–Whitney *U*‐test to compare between two groups; *p < 0.05; **p < 0.01; *p* < 0.001.AA, advanced adenoma; CRC, colorectal cancer; HP, hyperplastic polyp; NAA, nonadvanced adenoma; SSL, sessile serrated lesion.

To investigate variations in methylation levels between different groups, we conducted a comparative analysis of the methylation status of individual genes across patients belonging to the hyperplastic polyps (HP) + control group, nonadvanced adenoma (NAA), AA, and CRC group, while excluding those with SSLs (*n* = 3). To perform *SEPT9* methylation analysis, 12 additional samples (2 CRC, 2 AA, 6 NAA, 1 HP, and 1 control) were excluded due to non‐compliance with ddPCR quality criteria (>10,000 droplets per 20 μL PCR reaction). In total, 247 plasma samples from participants were analyzed for *SEPT9* methylation status (Table 2). Our findings indicate a statistically significant difference between groups (*p* = 0.047), with a higher median level of methylation status observed in the CRC group in comparison to the AA (*p* = 0.046), NAA (*p* = 0.017), and control/HP (*p* = 0.009) groups (Figure 3A). Further analysis revealed that *SEPT9* methylation status was significantly elevated in stage IV CRC in contrast to stages I (*p* = 0.0004), II (*p* = 0.004) and III (*p* = 0.0003) (Figure 3B).

**FIGURE 4:**
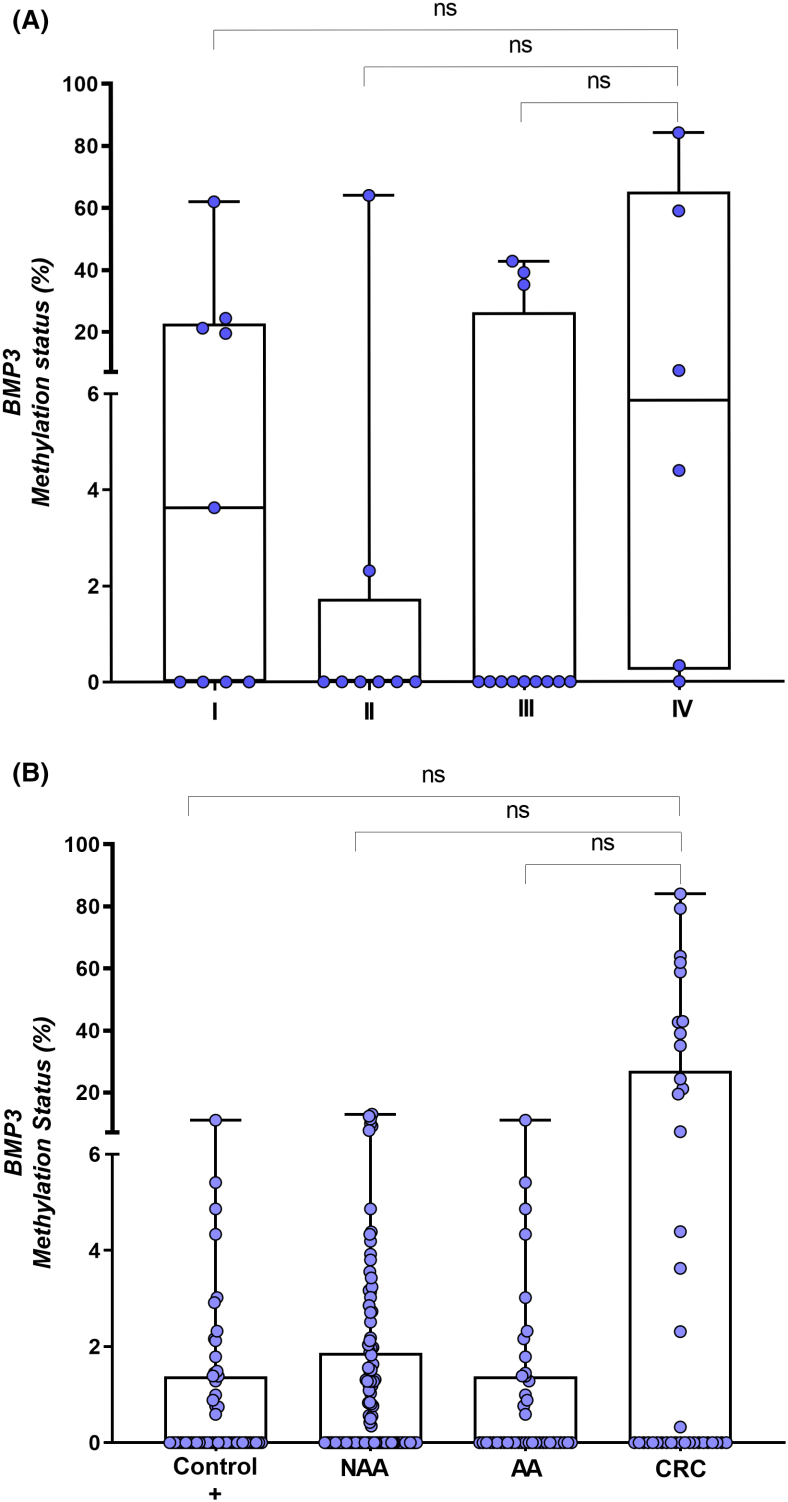
Methylation status of *BMP3* according to: (A) Lesion group (Kruskal–Wallis test; *p* = 0.128) and (B) Tumor stage (I–IV) (Kruskal–Wallis test; *p* = 0.151).HP, hyperplastic polyp; SSL, sessile serrated lesion; NAA, nonadvanced adenoma; AA, advanced adenoma; CRC, colorectal cancer; ns, not significant.

For *BMP3*methylation analysis, 15 samples (3 CRC, 1 AA, 9 NAA, 1 HP and 1 control) were excluded due to ddPCR quality criteria (also low number of droplets were measured). We ultimately analyzed 244 plasma samples (Table 2) and did not observe any significant differences between groups (*p* = 0.128) or tumor stage (*p* = 0.151) (Figure 4A,B).

### 3.4 Sensitivity and specificity of *SEPT9* and *BMP3* genes for advanced neoplasia detection

**FIGURE 5:**
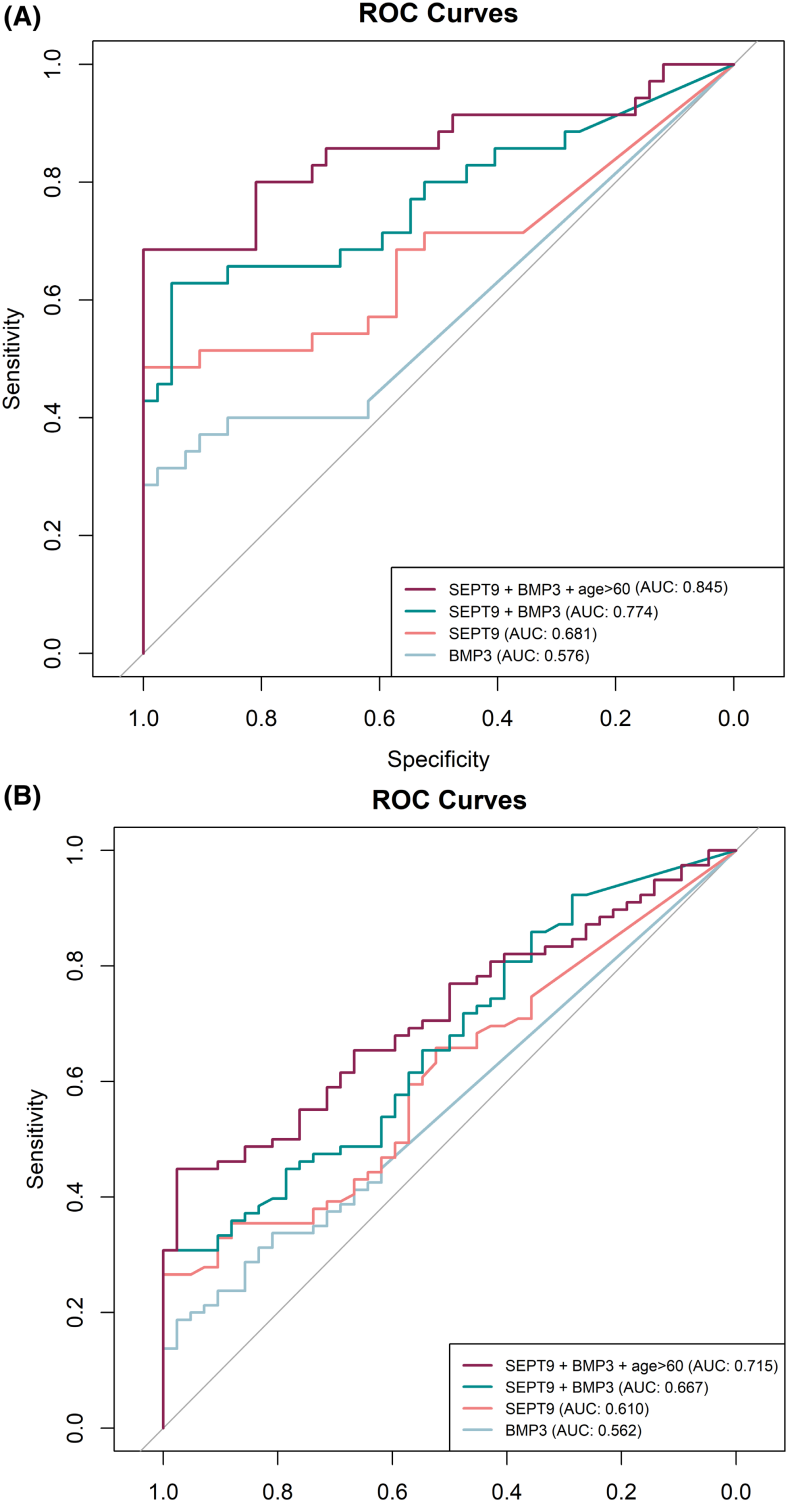
Comparison of ROC Curves (A) for CRC (*n* = 35) diagnostic of *SEPT9 (*AUC = 0.681; CI = 0.544–0.794) and *BMP3 (*AUC = 0.576; CI = 0.452–0.690) methylation. Combination of *SEPT9* + *BMP3* (AUC = 0.774; CI = 0.662–0.886) and *SEPT9* + *BMP3* + age > 60 (AUC = 0.845; CI = 0.767–0.943). (B) for Advanced adenoma (*n* = 44) + CRC (*n* = 35) diagnostic of *SEPT9 (*AUC = 0.610; CI = 0.510–0.710) and *BMP3 (*AUC = 0.562; CI = 0.470–0.654) methylation. Combination of *SEPT9* + *BMP3* (AUC = 0.667; CI = 0.569–0.765) and *SEPT9* + *BMP3* + age > 60 (AUC = 0.715; CI = 0.625–0.805). AUC, area under the curve. CI, confidence interval.

The area under the curve (AUC) for the *SEPT9* gene was 0.681, and a cutoff point of 2.5% of methylation status was set (Figure 5A). Using this cutoff value, the sensitivity and specificity for cancer detection were 50% and 90%, respectively. The positive detection rates for NAA and AA were 15.9% (18/113) and 16.0% (7/44), respectively, while the false‐positive rates were 9.5% for controls (4/42), 15.9% for NAA (18/113) and 15.9% for AA (7/44). The 2.5% cutoff value detected all stage IV CRCs (100%), compared to 22.2% of stage I CRC (Table 3).

**TABLE 3:** Detection rates in different *SEPT9* and *BMP3* methylation cutoff values.

*SEPT9* methylation–Cutoffs					
		0.84 (AA + CRC)		2.50 (CRC)	
		Negative (%)	Positive (%)	Negative (%)	Positive (%)
Lesion^a^ (*n* = 247)					
Control^a^	42	25 (59.5)	17 (40.5)	38 (90.5)	4 (9.5)
Adenomas					
NAA	113	48 (42.5)	65 (57.5)	95 (84.1)	18 (15.9)
AA	44	21 (47.7)	23 (52.3)	37 (84.0)	7 (16.0)
Serrated Polyp					
HP	12	7 (58.3)	5 (41.7)	12 (100.0)	0 (0.0)
Cancer	36	12 (33.4)	24 (66.6)	17 (47.3)	19 (52.7)
Stage (*n* = 36)					
I	9	5 (55.6)	4 (44.4)	7 (77.8)	2 (22.2)
II	8	3 (37.5)	5 (62.5)	4 (50.0)	4(50.0)
III	13	5 (38.5)	8 (61.5)	7 (53.8)	6 (46.2)
IV	6	0 (0.0)	6 (100.0)	0 (0.0)	6 (100.0)

*BMP3* methylation–Cutoffs					
		0.16 (AA + CR)		2.30 (CRC)	
		Negative (%)	Positive (%)	Negative (%)	Positive (%)
Lesion^a^ (*n* = 244)					
Control^a^	42	26 (61.9)	16 (38.1)	37 (88.1)	5 (11.9)
Adenomas					
NAA	110	60 (54.5)	50 (45.5)	90 (81.8)	20 (18.2)
AA	45	24 (53.3)	21 (46.7)	39 (86.7)	6 (13.3)
Serrated Polyp					
HP	12	8 (66.7)	4 (33.3)	11 (91.7)	1 (8.3)
Cancer	35	20 (57.1)	15 (42.9)	22 (62.9)	13 (37.1)
Stage (*n* = 35)					
I	9	4 (44.4)	5 (55.6)	4 (44.4)	5 (55.6)
II	8	6 (75.0)	2 (25.0)	7 (87.5)	1 (12.5)
III	12	9 (75.0)	3 (25.0)	9 (75.0)	3 (25.0)
IV	6	1 (16.7%)	5 (83.3)	2 (33.3)	4 (66.7)

*Note*: Chi‐squared test was used to calculate *p* value.

Abbreviations: AA, advanced adenoma; CRC, colorectal cancer; HP, hyperplastic polyp; NAA, nonadvanced adenoma.

^**a**^ Samples with no significant lesion identified on the colonoscopy.

Concerning the *BMP3* methylation status, the AUC for the model discriminating control group versus CRC group, was 0.576, and a cutoff point of 2.3% of *BMP3* methylation status was set with 40% of sensitivity and 90% of specificity for CRC detection (Figure 5A). Using this cutoff value, 18.2% (20/110) of NAAs and 13.3% (6/45) of AAs were detected. Furthermore, using the 2.3% cutoff value, 66.7% stage IV CRCs (4/6) were detected compared to 55.6% of stage I CRC (5/4) (Table 3).

In the model comparing AA + CRC versus control group, the *SEPT9* gene showed an AUC of 0.610. The optimal cutoff value determined was 0.84%, resulting in a sensitivity of 64% and a high false‐positive rate (specificity of 57%) in detecting advanced neoplasia (AA + CRC). At this cutoff value, the detection rates for NAA and AA were 57% (65/113) and 52.3% (23/44), respectively (Table 3, Figure 5B). Likewise, for the *BMP3* gene in the AA + CRC versus control model, the AUC was 0.562, and the optimal cutoff value for methylation status was 0.16%, resulting in 45% sensitivity and 62% specificity in the detection of advanced neoplasia (AA + CRC) (Figure 5B). The detection rates of NAA and AA were 45.5% (50/110) and 46.7% (21/45), respectively (Table 3).

### 3.5 Combination of DNA methylation markers for CRC and AA + CRC diagnosis

We further assessed whether combining *SEPT9* and *BMP3* promoter methylation improves the discrimination between CRC and control groups, as well as between AA + CRC versus control groups. The combination of these two markers showed better diagnostic performance to discriminate CRC and control groups, compared to either marker alone, with a sensitivity of 65.0% and a specificity of 86.0% (AUC = 0.774) (Figure 5A). In the discrimination between AA + CRC and control groups, the combined methylation markers achieved a sensitivity of 67.0% and a specificity of 52.0% (AUC = 0.667) (Figure 5B).

To further improve the discrimination of CRC patients from controls, we constructed a logistic model that combined *SEPT9* and *BMP3* methylation with age > 60. This model yielded superior results with an AUC of 0.845, a sensitivity of 80.0%, and a specificity of 81.0% (Figure 5A). Similarly, for AA + CRC versus control discrimination, the combination of *SEPT9* and *BMP3* methylation with age > 60 achieved a sensitivity of 74.0% and a specificity of 54.0% with an AUC of 0.715 (Figure 5B).

**FIGURE 6:**
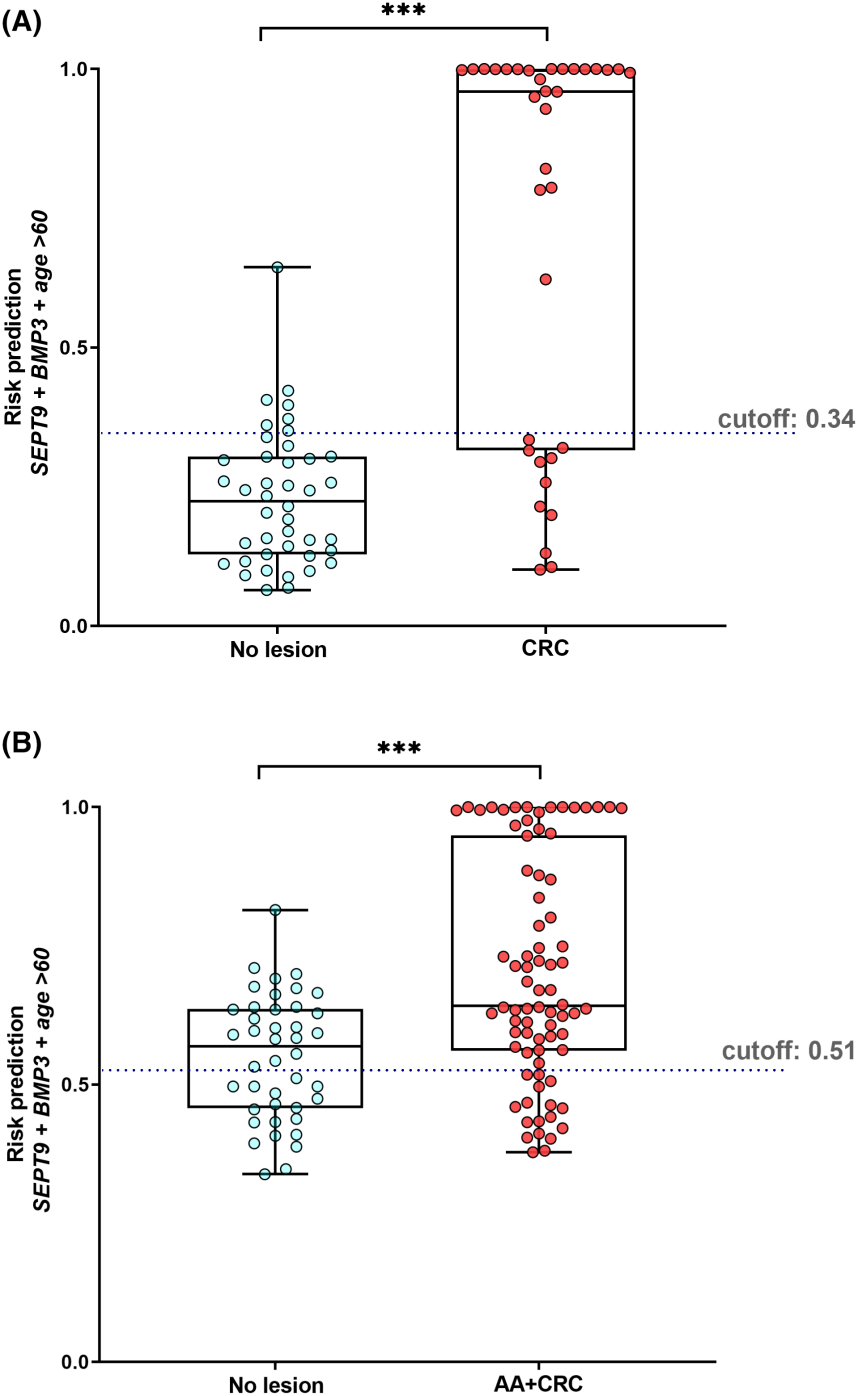
The risk score prediction. (A) no lesion group (Control group, *n* = 42) and CRC stage I–IV (*n* = 35) patients. (B) no lesion group (Control group, *n* = 42) and AA + CRC (*n* = 79) patients. Mann–Whitney *U*‐test to compare between two groups; ***p < 0.001.

The risk probabilities were calculated using Euclidean's index, and the distributions were plotted for the control, CRC, AA + CRC groups. The probability of CRC risk generated from the model for control and CRC groups resulted in a risk score threshold of 0.34 (Figure 6A). Similarly, the probability of AA + CRC risk generated from the model for control and AA + CRC groups resulted in a risk score threshold of 0.51 (Figure 6B).

## 4 DISCUSSION

In the present study, we developed an ultrasensitive methodology (ddPCR) to investigate cfDNA methylation status of *SEPT9* and *BMP3* in plasma from a series of 262 cases, including normal, precursor lesions and CRC lesions. The AUC accuracy for detecting CRC was 0.681 and 0.576 for *SEPT9* and *BMP3* markers, respectively. The combination of the two genes along with age > 60, significantly improved the diagnostic performance for the detection of CRC, with an AUC of 0.845. The diagnostic performance of both genes plus age > 60 was lower for discriminating high‐risk precursor lesion (AA), with and AUC of 0.715.

We found that screening participants with nonadvanced adenoma or SSL (NAA + SSL group) and CRC group showed the highest median values of circulating cfDNA in ng/mL of plasma compared to the control group (normal colonoscopy). Our results are in agreement with previous reports that showed a significant difference among cfDNA concentration from CRC patients and control individuals after quantification by fluorimetric methods.^37^, ^38^ Concerning NAA and AA groups, we did not notice difference in cfDNA levels concentration compared to patients with no colorectal lesion. Similarly, a previous study also compared NAA and AA with control group and did not detect significant difference on cfDNA concentration (ng/mL) between control, NAA and AA groups.^38^ Regarding different CRC stages, Stage IV patients exhibited the highest median value of cfDNA level, corroborating previous studies showing that cfDNA levels in CRC patients varies according to the cancer stage.^37^, ^38^, ^39^ However, it is worth mentioning that the cfDNA median level of AA screening participants was not different from those levels of normal colonoscopy participants or NAA+ SSL group.

Plasma *SEPT9* methylation has been extensively studied in CRC patients as a diagnostic or predicted treatment marker.^40^, ^41^ *SEPT9* encodes a GTP‐binding protein involved in several cellular processes such as cytoskeletal remodeling. So far, three *SEPT9* transcripts (v5, v4, and v2) are overexpressed in CRC epithelial cells compared to normal cells.^42^ The sensitivity of Epi proColon®, a qRT‐PCR‐based assay to detect methylated *SEPT9* in blood approved by both FDA for CRC screening is higher for more advanced colorectal neoplasia, being 11.2%, 35.0%, and 63.0% for AA, Stage I, and Stage II CRCs, respectively.^14^, ^24^ Several studies have investigated the diagnostic value of blood m*SEPT9* for CRC detection, with rather discrepant results of sensitivity and specificity, varying from 36.6% to 95.6% and 77.0% to 98.9%, respectively.^20^, ^21^, ^22^, ^23^, ^24^ In our study the ddPCR assay of *mSEPT9* yielded 50.0% of sensitivity and 90.0% of specificity for CRC. Our models had similar results to a previous study to detect *SEPT9* methylation in plasma by ddPCR.^41^ Zhi Yao Ma et al. reported a cutoff at 2.9 to distinguish CRC patients from controls with 73.7% of sensitivity but lower specificity (50%).^41^ A recent study also using ddPCR to detect *SEPT9* methylation in circulating cfDNA, showed 40.0% of sensitivity to detect NAA + AA and 55.6% for CRC, while the specificity was 80.0%.^43^

*BMP3* gene aberrant methylation has also been associated with initiation and progression of CRC.^44^ *BMP3* gene is a member of the transforming growth factor‐beta (TGFB) superfamily of cytokines, and usually is repressed during the early stages of most CRC cases.^45^ Therefore, *BMP3* methylation marker has been added to the Cologuard® test, a stool‐based test, FDA‐approved for CRC screening. This test showed 92.3% of sensitivity and 90.0% of specificity for CRC and 42.4% of sensitivity for AA.^46^ In our study, the *BMP3* methylation model yielded 40.0% of sensitivity and 90.0% of specificity for CRC.

It has been reported that the combination of biomarkers is a way to improve assay sensitivity while maintaining adequate specificity.^40^ In the present study, a combination model of two genes (*SEPT9* and *BMP3*) yielded 65.0% of sensitivity and 86.0% of specificity. In this line, Zhao et al. combined methylated *SEPT9* and *SDC2* in plasma by using ColoDefense® test, a new blood‐based methylation assay for CRC screening. The sensitivity and specificity of ColoDefense® test for CRC detection were 88.9% and 92.8%, respectively, higher than they found for *SEPT9* methylation alone (sensitivity of 65%).^44^ These findings provide valuable insights and support the rationale for considering the inclusion of *SDC2* methylation in combination with *SEPT9* and *BMP3* in future studies. Including *SDC2* in a multi‐marker panel may enhance the accuracy and sensitivity of the assay, thereby increasing its potential markers for improved diagnostic or screening assays for CRC. Future studies can focus on evaluating the performance and feasibility of such a combination model in our population to further validate its effectiveness in CRC detection or screening.

Based on a previous studies,^40^, ^47^, ^48^ we included the variable age > 60 (mean value between control and CRC groups ages) in the CRC and AA + CRC discrimination models. cfDNA combination model for CRC + age > 60 yielded 80.0% of sensitivity and 81.0% of specificity, significantly than the two individual assays (50% and 40%). A risk score threshold of 0.51, the cfDNA discrimination model for AA + CRC + age > 60 yielded 74.0% of sensitivity and 54.0% of specificity, indicating that the cfDNA methylation model including age > 60 could serve to improve the awareness of high‐risk subjects to achieve CRC screening.

Therefore, the combination of ddPCR assay and FIT could potentially increase the sensitivity of adenoma detection, especially for those with a high risk of CRC or those with an inconclusive positive FIT test. However, it is also important to note that this combination may lead to a reduction in specificity, which could result in unnecessary follow‐up colonoscopies, as has been seen with Cologuard® MT‐sDNAstool test.^46^ ddPCR could be particularly useful for triaging high‐risk FIT‐positive patients to undergo colonoscopy. The ddPCR assay can help to identify patients who have a higher risk of CRC and may benefit from further evaluation with colonoscopy. Nevertheless, further studies are needed to evaluate the clinical utility and cost‐effectiveness of using the ddPCR assay to triage high‐risk FIT‐positive patients for colonoscopy.

Despite the interesting findings, our study has some limitations. Firstly, the small number of samples from CRC group compared with samples from early adenoma group, AA and control group. Secondly, the present study only analyzed the methylation of two tumor suppressor genes, it is important to further investigate a large panel including other potential biomarkers.

In summary, using a sensitive technology (ddPCR), we showed that cfDNA *SEPT9* methylated marker is sensitive for advanced colorectal neoplasia detection (CRC + AA). Further, higher sensitivity and specificity for CRC detection when combined *SEPT9* with *BMP3* methylation, and even higher in participants aged >60. This is the first study of DNA based approach in liquid biopsy as a promise strategy for CRC screening and early diagnosis in a Brazilian population.

## AUTHOR CONTRIBUTIONS

**Adhara Brandão Lima:** Formal analysis (equal); investigation (equal); methodology (equal); validation (equal); writing – original draft (equal). **Mariana Bisarro dos Reis:** Data curation (equal); formal analysis (equal); investigation (equal); methodology (equal); validation (equal); writing – review and editing (equal). **Marcus Matsuhita:** Data curation (equal). **Monise Tadin:** Data curation (equal). **Marco Antônio de Oliveira:** Formal analysis (equal); software (equal); visualization (supporting). **Rui Manuel dos Reis:** Conceptualization (equal); data curation (equal); project administration (equal); supervision (equal); validation (equal); writing – original draft (equal); writing – review and editing (equal). **Denise Peixoto Guimarães:** Conceptualization (lead); data curation (equal); methodology (equal); supervision (lead); writing – original draft (equal); writing – review and editing (equal).

## FUNDING INFORMATION

Barretos Cancer Hospital and the Public Ministry of Labor Campinas supported this study. RMR was funded by the National Council for Scientific and Technological Development (CNPq, Brazil) as Research Productivity Scholarship ‐ Level 1B. Coordination for the improvement of Higher Education Personnel (CAPES, Brazil) funded A.B.L. and M.B.R.

## CONFLICT OF INTEREST STATEMENT

The authors have no conflicts of interest.

## Ethics approval and consent to participate

Research Ethical Committee of Barretos Cancer Hospital approved the study protocol (number ID: 3.285.683). All study participants, before the sample collection and participation, signed written informed consent.

## Supporting information


Table S1.

Figure S1.

## Data Availability

The data that support the findings of this study are available within the Supporting Information files and from the corresponding author upon request.

## References

[1] Sung H , Ferlay J , Siegel RL , et al. Global cancer statistics 2020: GLOBOCAN estimates of incidence and mortality worldwide for 36 cancers in 185 countries. CA Cancer J Clin. 2021;71(3):209‐249. 33538338 10.3322/caac.21660

[2] Instituto Nacional de Câncer (Brasil) . Estimativa 2023: incidência de câncer no Brasil. 2022 https://www.inca.gov.br/sites/ufu.sti.inca.local/files/media/document/estimativa‐2023.pdf

[3] Siegel RL , Wagle NS , Cercek A , Smith RA , Jemal A . Colorectal cancer statistics, 2020. CA Cancer J Clin. 2020;70(3):145‐164. 32133645 10.3322/caac.21601

[4] Crosby D , Bhatia S , Brindle KM , et al. Early detection of cancer. Science. 2022;375:6586. 10.1126/science.aay9040 35298272

[5] Digestive System Tumours . WHO Classification of Tumours. Vol 1. 5th ed. World Health Organization; 2019.

[6] Yamane L , Scapulatempo‐Neto C , Reis RM , Guimaraes DP . Serrated pathway in colorectal carcinogenesis. World J Gastroenterol. 2014;20(10):2634‐2640. 24627599 10.3748/wjg.v20.i10.2634 PMC3949272

[7] Winawer SJ , Zauber AG , Ho MN , et al. Prevention of colorectal cancer by Colonoscopic polypectomy. N Engl J Med. 1993;329(27):1977‐1981. 8247072 10.1056/NEJM199312303292701

[8] Schreuders EH , Ruco A , Rabeneck L , et al. Colorectal cancer screening: a global overview of existing programmes. Gut. 2015;64(10):1637‐1649. 26041752 10.1136/gutjnl-2014-309086

[9] Tepus M , Yau TO . Non‐invasive colorectal cancer screening: an overview. Gastrointestinal Tumors. 2020;7(3):62‐73. 32903904 10.1159/000507701 PMC7445682

[10] Lewis JM , Heineck DP , Heller MJ . Detecting cancer biomarkers in blood: challenges for new molecular diagnostic and point‐of‐care tests using cell‐free nucleic acids. Expert Rev Mol Diagn. 2015;15(9):1187‐1200. 26189641 10.1586/14737159.2015.1069709

[11] Jones PA , Baylin SB . The fundamental role of epigenetic events in cancer. Nat Rev Genet. 2002;3(6):415‐428. 12042769 10.1038/nrg816

[12] Sharma S , Kelly TK , Jones PA . Epigenetics in cancer. Carcinogenesis. 2010;31(1):27‐36. 19752007 10.1093/carcin/bgp220 PMC2802667

[13] Jung G , Hernández‐Illán E , Moreira L , Balaguer F , Goel A . Epigenetics of colorectal cancer: biomarker and therapeutic potential. Nat Rev Gastroenterol Hepatol. 2020;17(2):111‐130. 31900466 10.1038/s41575-019-0230-y PMC7228650

[14] Payne SR . From discovery to the clinic: the novel DNA methylation biomarker m SEPT9 for the detection of colorectal cancer in blood. Epigenomics. 2010;2(4):575‐585. 22121975 10.2217/epi.10.35

[15] Sobanski T , Arantes LMRB , Dos Santos W , et al. Methylation profile of colon cancer genes in colorectal precursor lesions and tumor tissue: perspectives for screening. Scand J Gastroenterol. 2021;56(8):920‐928. 34218733 10.1080/00365521.2021.1922744

[16] Lofton‐Day C , Model F , Devos T , et al. DNA methylation biomarkers for blood‐based colorectal cancer screening. Clin Chem. 2008;54(2):414‐423. 18089654 10.1373/clinchem.2007.095992

[17] deVos T , Tetzner R , Model F , et al. Circulating methylated SEPT9 DNA in plasma is a biomarker for colorectal cancer. Clin Chem. 2009;55(7):1337‐1346. 19406918 10.1373/clinchem.2008.115808

[18] Dublin Pathology 2015. 8th joint meeting of the British division of the international academy of pathology and the pathological Society of Great Britain & Ireland, 23‐25 June 2015. J Pathol. 2015;237:S1‐S52. 26373699 10.1002/path.4631 PMC7168113

[19] Berger BM , Levin B , Hilsden RJ . Multitarget stool DNA for colorectal cancer screening: a review and commentary on the United States preventive services draft guidelines. World J Gastrointest Oncol. 2016;8(5):450. 27190584 10.4251/wjgo.v8.i5.450 PMC4865712

[20] Tóth K , Sipos F , Kalmár A , et al. Detection of methylated SEPT9 in plasma is a reliable screening method for both left‐ and right‐sided colon cancers. PLoS One. 2012;7(9):e46000. 23049919 10.1371/journal.pone.0046000 PMC3457959

[21] Lee HS , Hwang SM , Kim TS , et al. Circulating methylated Septin 9 nucleic acid in the plasma of patients with gastrointestinal cancer in the stomach and colon. Transl Oncol. 2013;6(3):290‐296. 23730408 10.1593/tlo.13118 PMC3660797

[22] Warren JD , Xiong W , Bunker AM , et al. Septin 9 methylated DNA is a sensitive and specific blood test for colorectal cancer. BMC Med. 2011;9:133. 22168215 10.1186/1741-7015-9-133 PMC3271041

[23] Ahlquist DA , Taylor WR , Mahoney DW , et al. The stool DNA test is more accurate than the plasma Septin 9 test in detecting colorectal neoplasia. Clin Gastroenterol Hepatol. 2012;10(3):272‐277.e1. 22019796 10.1016/j.cgh.2011.10.008 PMC3980432

[24] Church TR , Wandell M , Lofton‐Day C , et al. Prospective evaluation of methylated SEPT9 in plasma for detection of asymptomatic colorectal cancer. Gut. 2014;63(2):317‐325. 23408352 10.1136/gutjnl-2012-304149 PMC3913123

[25] Fatumo S , Chikowore T , Choudhury A , et al. A roadmap to increase diversity in genomic studies. Nat Med. 2022;28(2):243‐250. 35145307 10.1038/s41591-021-01672-4 PMC7614889

[26] Guimarães DP , Mantuan LA , de Oliveira MA , et al. The performance of colorectal cancer screening in Brazil: the first two years of the implementation program in Barretos cancer hospital. Cancer Prev Res. 2021;14(2):241‐252. 10.1158/1940-6207.CAPR-20-0179 32998941

[27] Participants in the Paris Workshop . The Paris endoscopic classification of superficial neoplastic lesions: esophagus, stomach, and colon. Gastrointest Endosc. 2003;58(6):S3‐S43. 14652541 10.1016/s0016-5107(03)02159-x

[28] Amin MB et al. AJCC Cancer Staging Manual (8th Edition). Springer International Publishing; 2017.

[29] Neuber AC , Tostes CH , Ribeiro AG , et al. The biobank of Barretos cancer hospital: 14 years of experience in cancer research. Cell Tissue Bank. 2022;23(2):271–284. 34216325 10.1007/s10561-021-09941-9

[30] Raymond C . Focused size selection of cell‐free DNA samples for liquid biopsy applications that rely on next‐generation sequencing. BioTechniques. 2019;67(4):188–191. 31502470 10.2144/btn-2019-0071

[31] Pharo HD , Andresen K , Berg KCG , et al. A robust internal control for high‐precision DNA methylation analyses by droplet digital PCR. Clin Epigenetics. 2018;10(1):24. 29484034 10.1186/s13148-018-0456-5 PMC5822558

[32] Litterst C , Shelton D , Patil M , Marrs S . Droplet digital™ PCR: detection of DNA methylation. Bulletin 6554 Rev A. 2014. https://www.biorad.com/webroot/web/pdf/lsr/literature/Bulletin_6554.pdf Accessed october 21, 2020.

[33] Bio‐Rad Laboratories . Droplet Digital PCR Applications Guide. http://www.bio‐rad.com/webroot/web/pdf/lsr/literature/Bulletin_6407.pdf Accessed september 08, 2020.

[34] dMIQE Group , Huggett JF . The digital MIQE guidelines update: minimum information for publication of quantitative digital PCR experiments for 2020. Clin Chem. 2020;66(8):1012‐1029. 32746458 10.1093/clinchem/hvaa125

[35] Clinical and Laboratory Standards Institute . Protocols for Determination of Limits of Detection and Limits of Quantitation, Approved Guideline. 2rd ed. Clinical and Laboratory Standards Institute, PA/USA; 2012.

[36] Armbruster DA , Pry T . Limit of Blank, limit of detection and limit of quantitation. Clin Biochem Rev. 2008;29 Suppl 1(Suppl 1):S49–52. 18852857 PMC2556583

[37] Frattini M , Gallino G , Signoroni S , et al. Quantitative and qualitative characterization of plasma DNA identifies primary and recurrent colorectal cancer. Cancer Lett. 2008;263(2):170‐181. 18395974 10.1016/j.canlet.2008.03.021

[38] Junca A , Tachon G , Evrard C , et al. Detection of colorectal cancer and advanced adenoma by liquid biopsy (Decalib study): the ddPCR challenge. Cancer. 2020;12(6):1482. 10.3390/cancers12061482 PMC7352444 32517177

[39] Boni L , Cassinotti E , Canziani M , Dionigi G , Rovera F , Dionigi R . Free circulating DNA as possible tumour marker in colorectal cancer. Surg Oncol. 2007;16:29‐31. 10.1016/j.suronc.2007.10.004 18024018

[40] Rasmussen SL , Krarup HB , Sunesen KG , et al. Hypermethylated DNA, a circulating biomarker for colorectal cancer detection. PLoS One. 2017;12(7):e0180809. 28700744 10.1371/journal.pone.0180809 PMC5507256

[41] Ma ZY , Chan CSY , Lau KS , Ng L , Cheng YY , Leung WK . Application of droplet digital polymerase chain reaction of plasma methylated septin 9 on detection and early monitoring of colorectal cancer. Sci Rep. 2021;11:23446. 34873218 10.1038/s41598-021-02879-8 PMC8648834

[42] Wasserkort R , Kalmar A , Valcz G , et al. Aberrant septin 9 DNA methylation in colorectal cancer is restricted to a single CpG Island. BMC Cancer. 2013;13:398. 23988185 10.1186/1471-2407-13-398 PMC3837632

[43] Suehiro Y , Hashimoto S , Higaki S , et al. Blood free‐circulating DNA testing by highly sensitive methylation assay to diagnose colorectal neoplasias. Oncotarget. 2018;9(24):16974‐16987. 29682198 10.18632/oncotarget.24768 PMC5908299

[44] Zhao G , Li H , Yang Z , et al. Multiplex methylated DNA testing in plasma with high sensitivity and specificity for colorectal cancer screening. Cancer Med. 2019;8(12):5619‐5628. 31407497 10.1002/cam4.2475 PMC6745865

[45] Loh K , Chia JA , Greco S , et al. Bone morphogenic protein 3 inactivation is an early and frequent event in colorectal cancer development. Genes Chromosomes Cancer. 2008;47(6):449‐460. 18311777 10.1002/gcc.20552

[46] Imperiale TF , Ransohoff DF , Itzkowitz SH , et al. Multitarget stool DNA testing for colorectal‐cancer screening. N Engl J Med. 2014;370(14):1287‐1297. 24645800 10.1056/NEJMoa1311194

[47] Vanaclocha‐Espi M , Ibáñez J , Molina‐Barceló A , et al. Optimal cut‐off value for detecting colorectal cancer with fecal immunochemical tests according to age and sex. PLoS One. 2021;16(7):e0254021. 34270590 10.1371/journal.pone.0254021 PMC8284629

[48] Alvarez‐Urturi C , Andreu M , Hernandez C , et al. Impact of age‐ and gender‐specific cut‐off values for the fecal immunochemical test for hemoglobin in colorectal cancer screening. Dig Liver Dis. 2016;48(5):542‐551. 26936343 10.1016/j.dld.2016.02.001

