# Supplementary material for: Combined SEPT9 and BMP3 methylation in plasma for colorectal cancer early detection and screening in a Brazilian population
Source: Cancer Med. 2023 Jun 20;12(15):15854–67. doi: 10.1002/cam4.6224 (PMC10469661; doi:10.1002/cam4.6224)
Supplement: Supplementary file 1 — Table S1. [file CAM4-12-15854-s001.docx]

Supplementary Table 1: Sequences of primers and probes, product sizes, and the number of CpG sites.

| **Gene** | **Primers e probes (5´- 3´)** | **Product** | **CpG sites** | **Amplicon mapping** (GRCh38/hg38) |
| --- | --- | --- | --- | --- |
| ***SEPT9 v2*** | Forward: AAATAATCCCATCCAACTA  Reverse: GTTGTTTATTAGTTATTATGT  Methylated probe: (FAM) TTAACCGCGAAATCCGAC  Unmethylated probe: (HEX) TTAACCACAAAATCCAACATAAT | 60 bp | 3 | chr17:77,373,481-77,373,540 |
| ***BMP3*** | Forward: GTAGTAAGTGGGGTTGGT  Reverse: ATTAAACTCCAAACCAACTAAAAC  Methylated probe: (FAM) TTGTATTCGGTCGCGTTTC  Unmethylated probe: (HEX) TTGTATTTGGTTGTGTTTTGGG | 88 bp | 3 | chr4:81,031,113-81,031,200 |

Supplementary Table 2: Numbers of copies/reaction obtained for calculation of Limit of Blank for 20 replicates for each gene analyzed.

| ***SEPT9* – LOB assay** | | ***BMP3* – LOB assay** | |
| --- | --- | --- | --- |
| **Methylated probe** | **Unmethylated probe** | **Methylated probe** | **Unmethylated probe** |
| 0 | 0 | 0 | 22.4 |
| 0 | 0 | 0 | 24.8 |
| 0 | 0 | 0 | 31.5 |
| 0 | 0 | 0 | 38.1 |
| 0 | 0 | 1.5 | 22.4 |
| 0 | 0 | 0 | 16.1 |
| 0 | 0 | 0 | 3.7 |
| 0 | 1 | 0 | 1.67 |
| 0 | 0 | 0 | 3.1 |
| 0 | 0 | 0 | 9.5 |
| 0 | 0 | 0 | 10 |
| 0 | 1 | 0 | 7.4 |
| 0 | 2 | 0 | 15.2 |
| 1.5 | 0 | 1.6 | 7.3 |
| 0 | 1 | 0 | 5.9 |
| 0 | 0 | 0 | 9.8 |
| 1.4 | 0 | 0 | 6.5 |
| 0 | 0 | 0 | 12.6 |
| 0 | 0 | 0 | 0 |
| 0 | 0 | 0 | 16.6 |
