# Supplementary material for: Combined SEPT9 and BMP3 methylation in plasma for colorectal cancer early detection and screening in a Brazilian population
Source: Cancer Med. 2023 Jun 20;12(15):15854–67. doi: 10.1002/cam4.6224 (PMC10469661; doi:10.1002/cam4.6224)
Supplement: Supplementary file 2 — Figure S1. [file CAM4-12-15854-s002.docx]

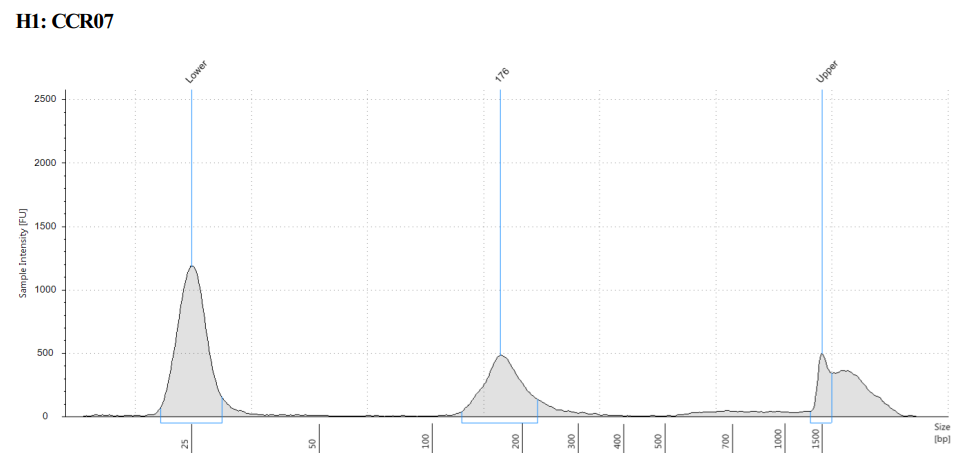


1. **Sample: CCR07**


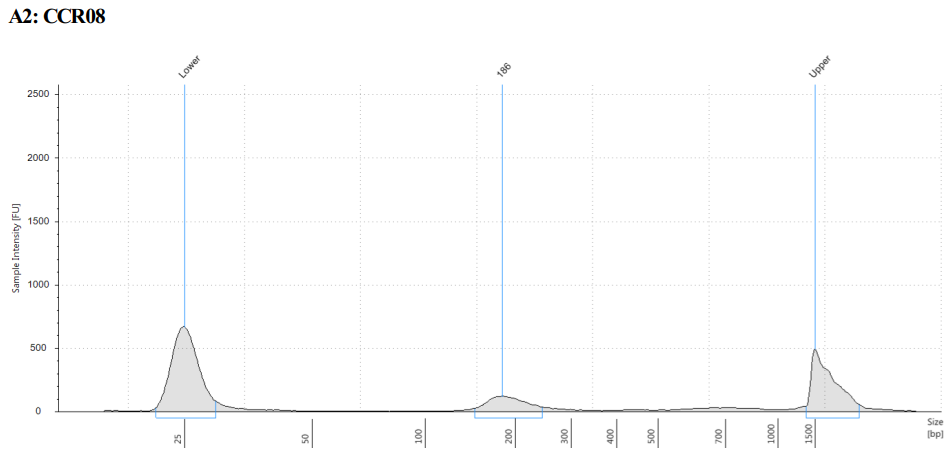


**b) Sample: CCR08**

**Supplementary Figure 1**


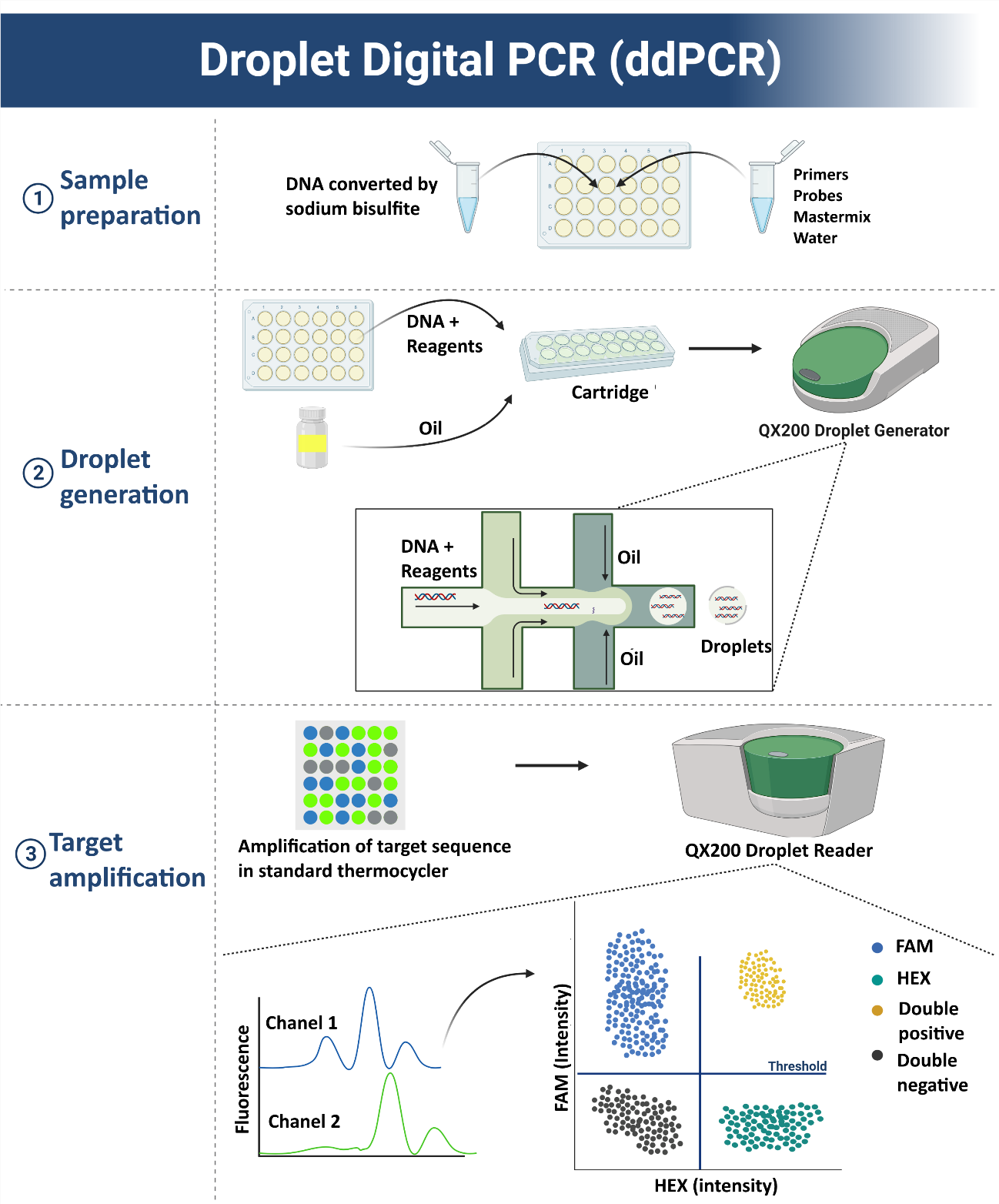


**Supplementary Figure 2**


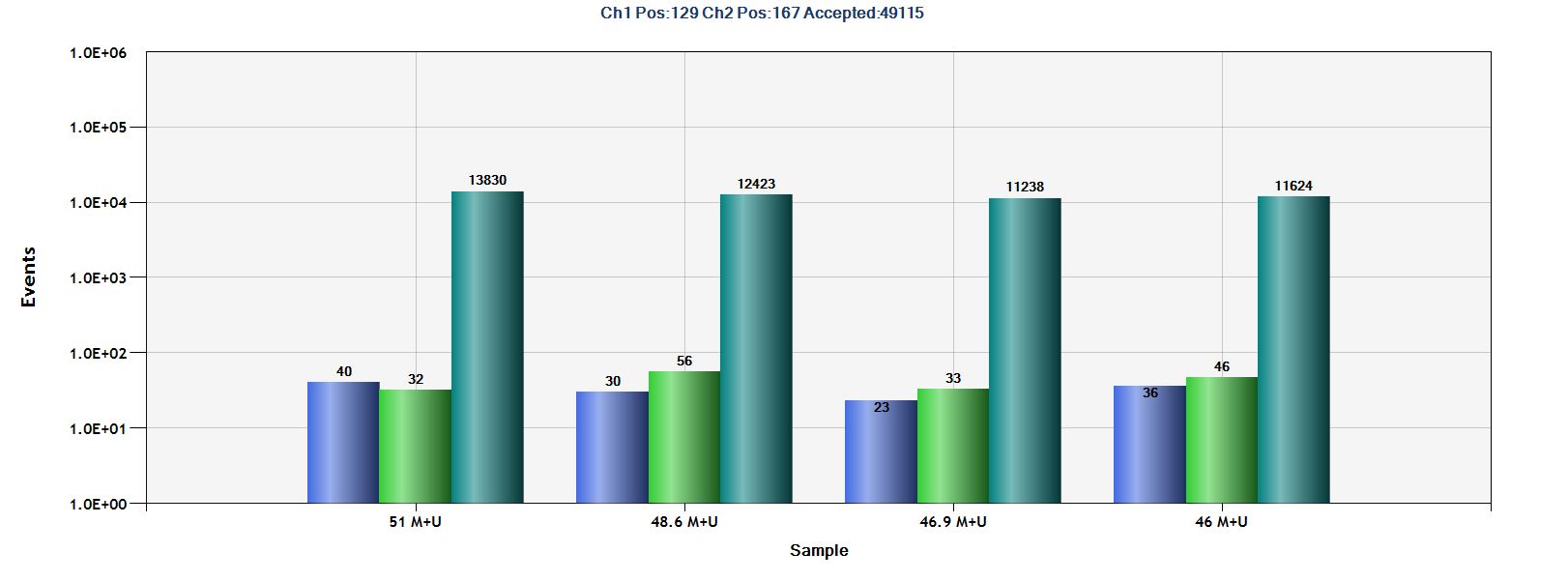


**b)**

**a)**

**
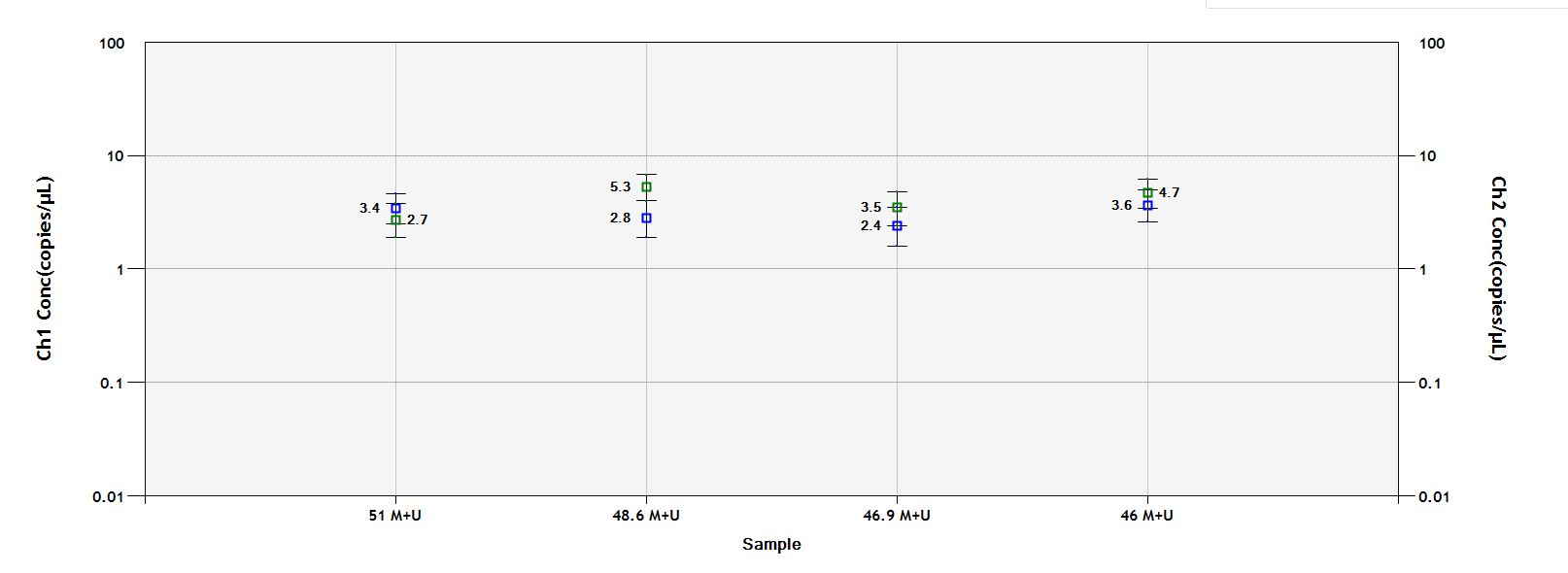
**

**c)**

**
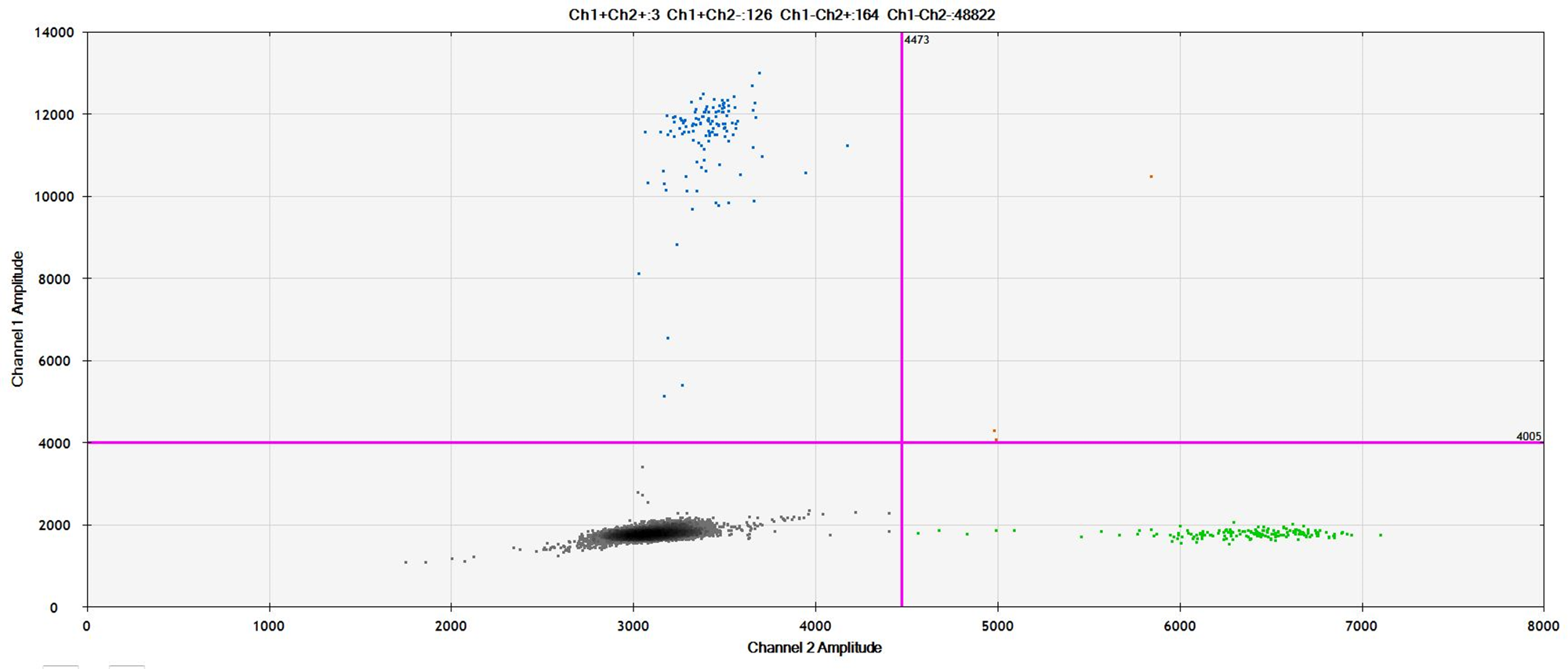
**

**Supplementary Figure 3**

**
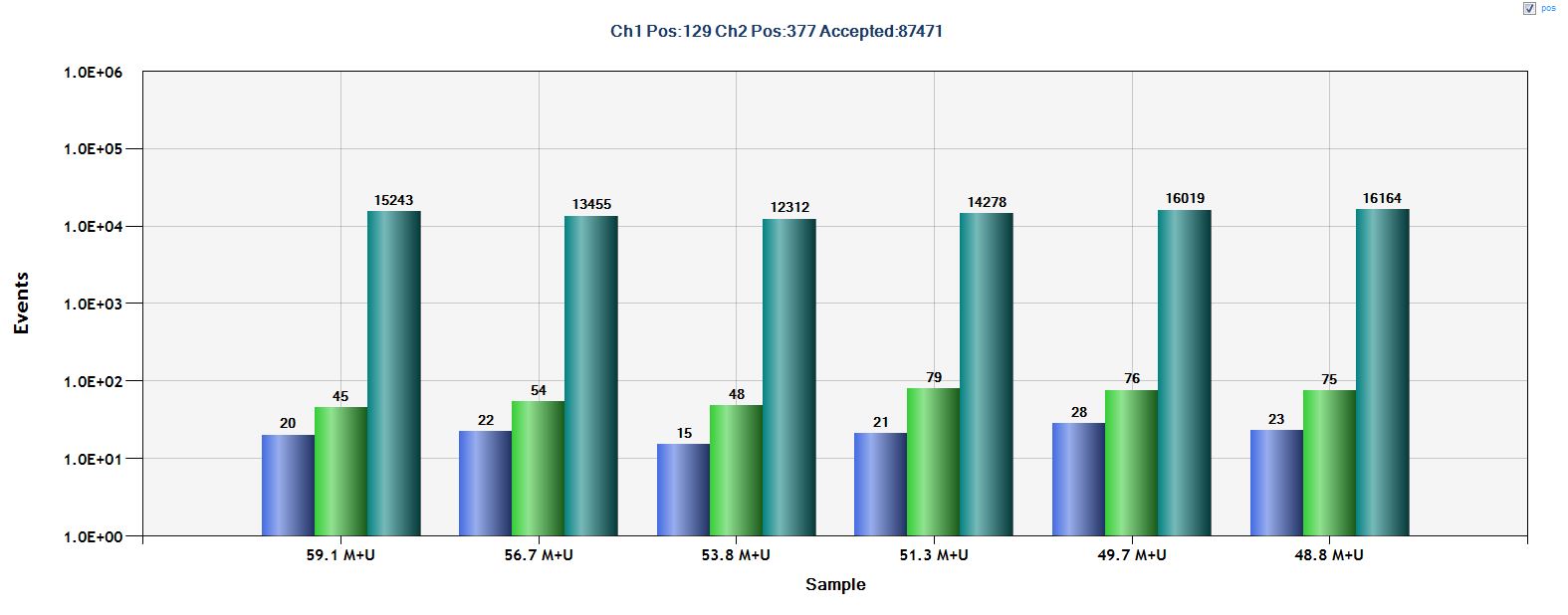
**

**b)**

**a)**

**
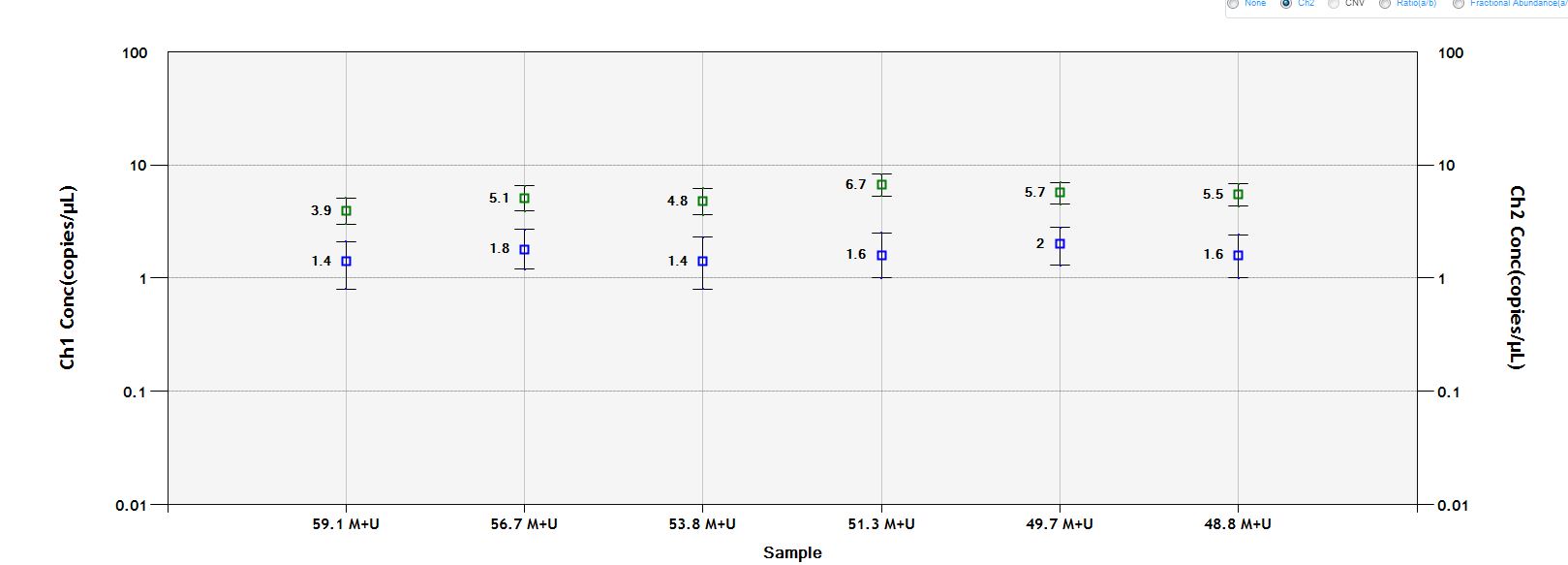
**

**c)**

**
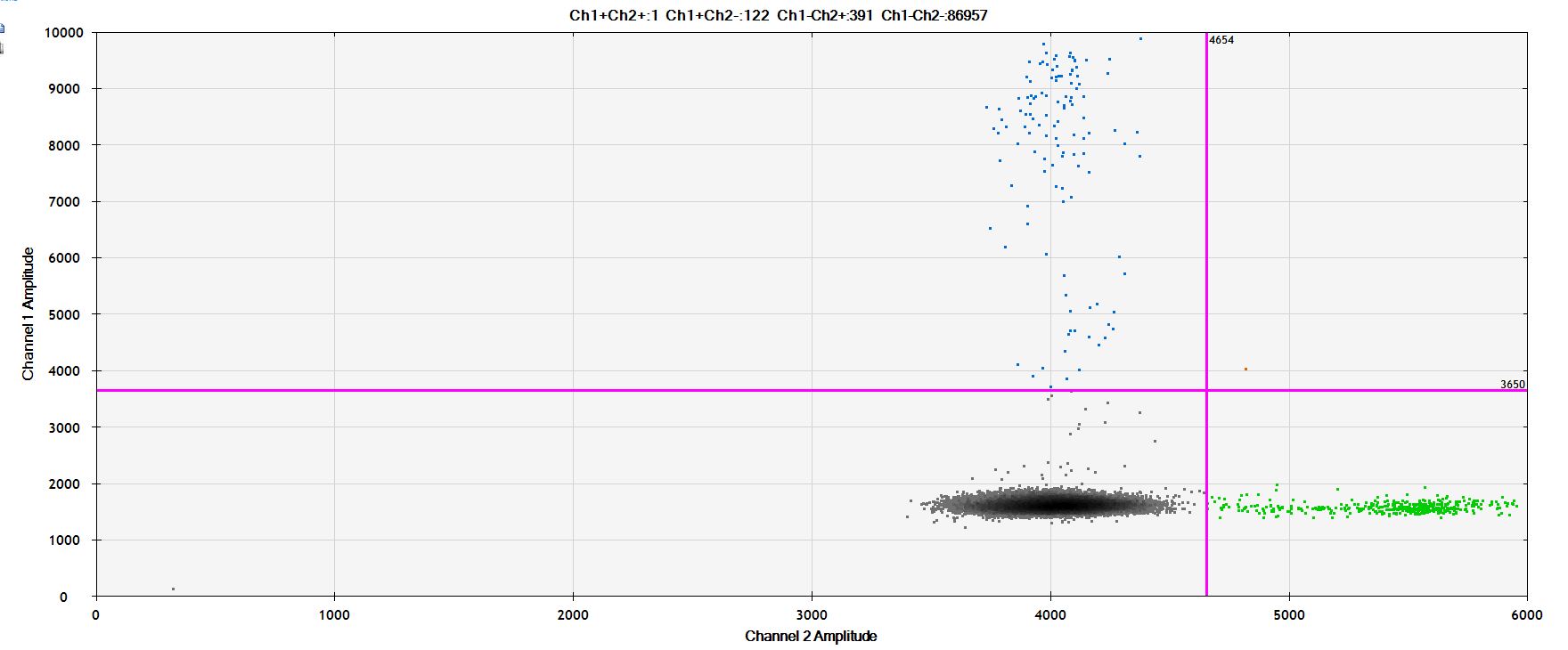
**

**Supplementary Figure 4**


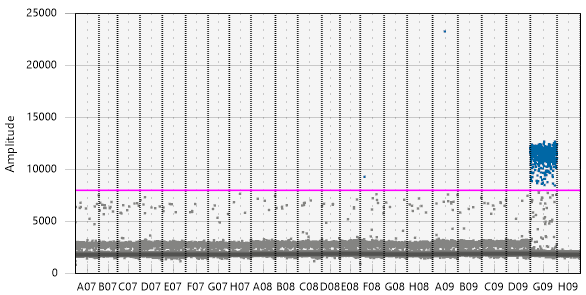


**C+**

**NTC**

**a)**

**b)**


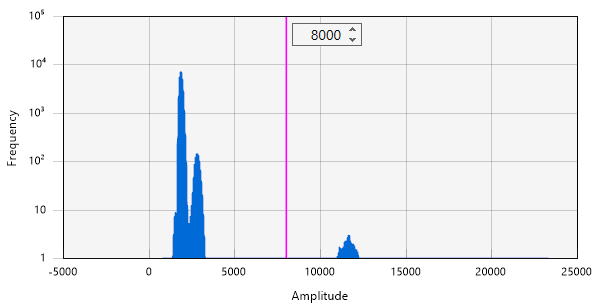


**C+**

**NTC**

**c)**


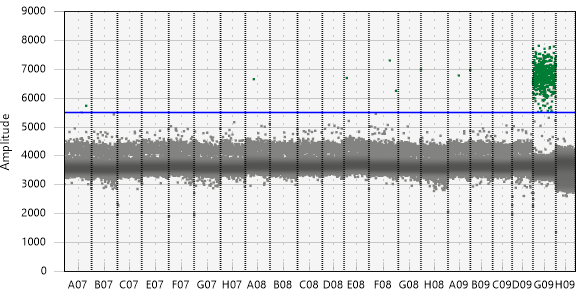


**d)**


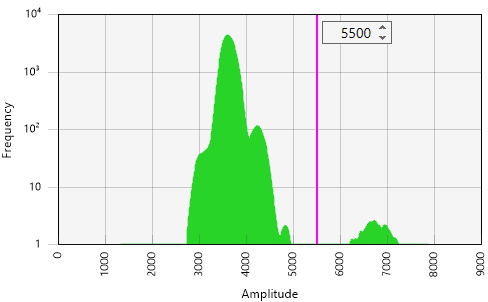


**Supplementary Figure 5**


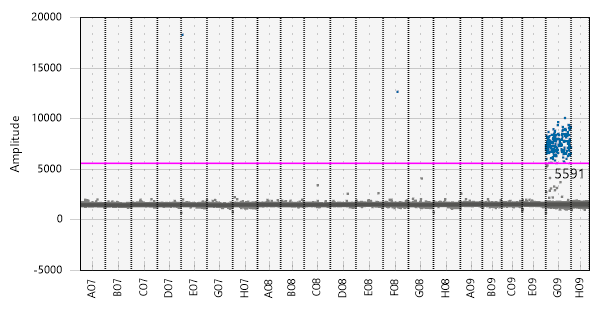


**NTC**

**C+**

**b)**

**c)**

**a)**


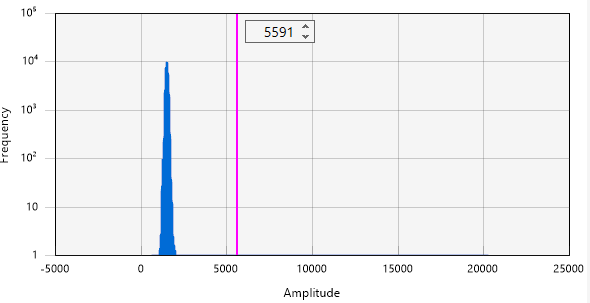


**C+**

**NTC**


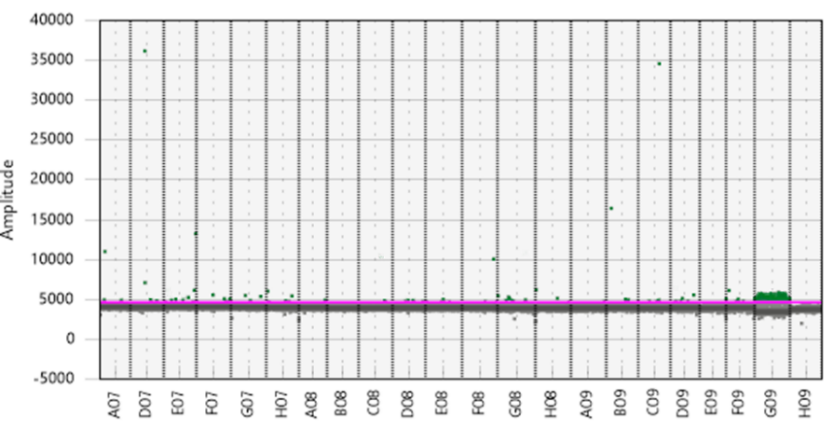

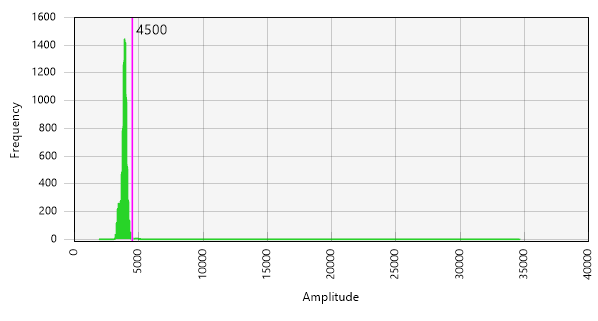


**d)**

**Supplementary Figure 6**

**a)**

**0.01ng**

**0.1ng**

**1ng**

**10ng**


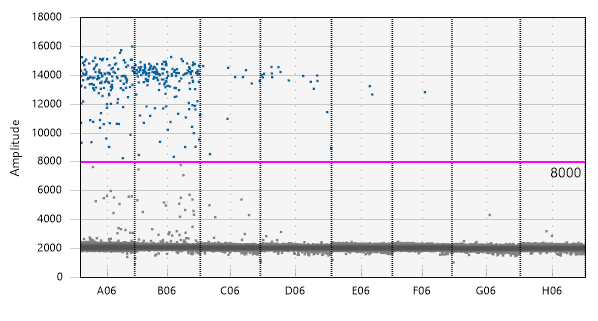


**0.1ng**

**1ng**

**0.01ng**

**b)**

**10ng**


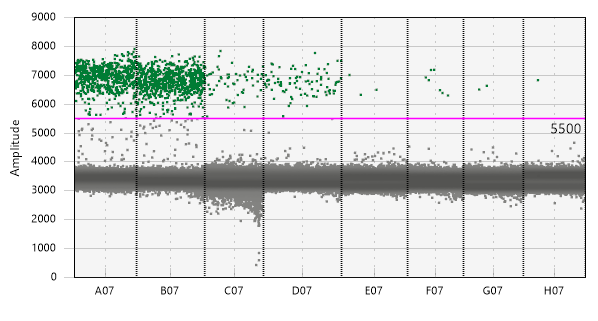


**Supplementary Figure 7**


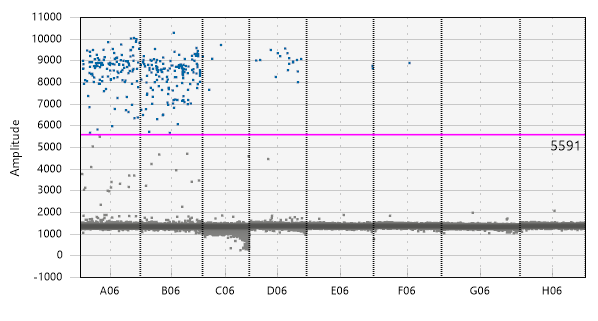


**b)**

**a)**

**0.1ng**

**1ng**

**10ng**

**0.01ng**

**0.1ng**

**10ng**

**1ng**

**0.01ng**


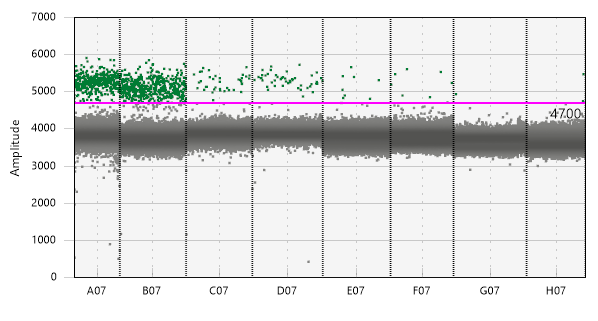


**Supplementary Figure 8**


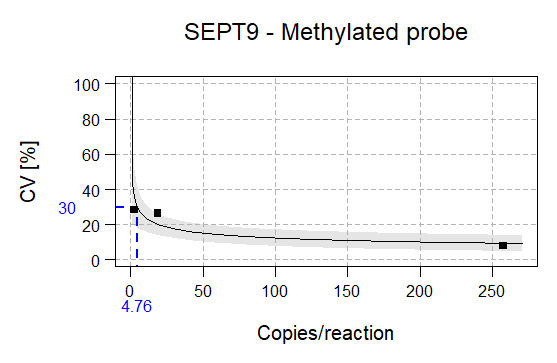

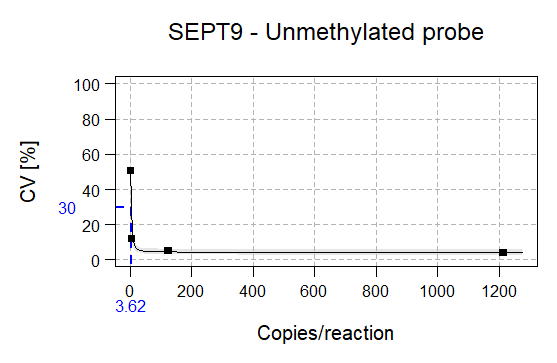

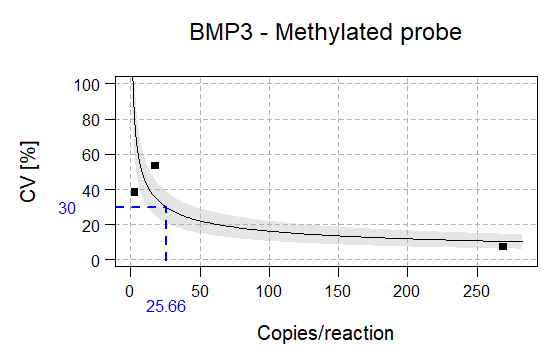

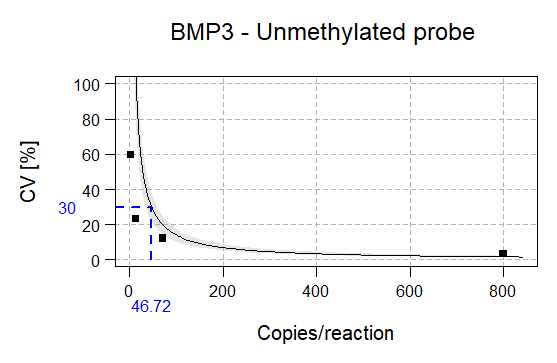


**c)**

**a)**

**b)**

**d)**

**Supplementary Figure 9**
